# Metabolite fingerprinting of pennycress (*Thlaspi arvense* L.) embryos to assess active pathways during oil synthesis

**DOI:** 10.1093/jxb/erv020

**Published:** 2015-02-22

**Authors:** Enkhtuul Tsogtbaatar, Jean-Christophe Cocuron, Marcos Corchado Sonera, Ana Paula Alonso

**Affiliations:** ^1^The Ohio State University, Department of Molecular Genetics, Columbus, OH 43210, USA; ^2^The Ohio State University, Center for Applied Plant Sciences, Columbus, OH 43210, USA; ^3^University of Puerto Rico, Mechanical Engineering Department, Mayagüez, 00681-9000, Puerto Rico

**Keywords:** Alternative crop, erucic acid, GC-MS, jet fuel, LC-MS/MS, metabolomics, oilseed, pennycress, plant metabolism, *Thlaspi arvense* L., triacylglycerols.

## Abstract

Pennycress (*Thlaspi arvense* L.), a plant naturalized to North America, accumulates high levels of erucic acid in its seeds, which makes it a promising biodiesel and industrial crop. The main carbon sinks in pennycress embryos were found to be proteins, fatty acids, and cell wall, which respectively represented 38.5, 33.2, and 27.0% of the biomass at 21 days after pollination. Erucic acid reached a maximum of 36% of the total fatty acids. Together these results indicate that total oil and erucic acid contents could be increased to boost the economic competitiveness of this crop. Understanding the biochemical basis of oil synthesis in pennycress embryos is therefore timely and relevant to guide future breeding and/or metabolic engineering efforts. For this purpose, a combination of metabolomics approaches was conducted to assess the active biochemical pathways during oil synthesis. First, gas chromatography–mass spectrometry (GC-MS) profiling of intracellular metabolites highlighted three main families of compounds: organic acids, amino acids, and sugars/sugar alcohols. Secondly, these intermediates were quantified in developing pennycress embryos by liquid chromatography–tandem mass spectrometry (LC-MS/MS) in multiple reaction monitoring mode. Finally, partitional clustering analysis grouped the intracellular metabolites that shared a similar pattern of accumulation over time into eight clusters. This study underlined that: (i) sucrose might be stored rather than cleaved into hexoses; (ii) glucose and glutamine would be the main sources of carbon and nitrogen, respectively; and (iii) glycolysis, the oxidative pentose phosphate pathway, the tricarboxylic acid cycle, and the Calvin cycle were active in developing pennycress embryos.

## Highlight

Metabolomics was shown to be a powerful tool for both quantifying intracellular compounds and qualitatively assessing biochemical pathways. This approach underlined metabolic routes involved in the synthesis of biofuel-relevant oils.

## Introduction

Petroleum is the largest energy source in the USA, accounting for 28% of all energy consumed in 2013 (www.eia.gov). The fact that petroleum-based fuels will eventually be depleted requires the development of renewable fuels. Indeed, biofuel production has attracted considerable research attention in both developing and industrialized countries (Demirbas, 2009). In addition to its ability to mitigate the approaching shortage of petroleum, renewable energy has the additional environmental benefit of being a low contributor of greenhouse gases (Kim and Dale, 2005). Biofuel is a renewable fuel that can be produced from plant biomass components such as oil, starch, and cell wall. In the USA, the most common biofuels, such as ethanol and biodiesel, are currently produced from corn, soybean, and other high cost commodity crops (Kim and Dale, 2005; Demirbas, 2009; Moser *et al.*, 2009*a*). In fact, it seems crucial to address that the biofuel industry should not use crops with valuable food applications. However, the availability of other suitable bioenergy plants, such as sugarcane, is limited to certain geographies and climates. Taking these challenges into consideration, the biofuel industry is in need of alternative crops that meet the following criteria: (i) a favourable biomass composition for biofuel production; (ii) the ability to grow in a variety of soils and climates; and (iii) have no competition with food crops. The alternative bioenergy crops that have been studied so far include, but are not restricted to, *Crambe* (Li *et al.*, 2012), *Camelina* (Frohlich and Rice, 2005), *Brassica carinata* (Bouaid *et al.*, 2009), *Miscanthus* (Robson *et al.*, 2013), and sugarcane (Hojilla-Evangelista *et al.*, 2013).

Field pennycress (*Thlaspi arvense* L.; Supplementary Fig. S1 available at *JXB* online) is a winter annual that grows widely across temperate regions of North America and the southern hemisphere (Warwick *et al.*, 2002). It has been identified as an oilseed crop that could be a suitable source for biofuel (Vaughn *et al.*, 2005; Hojilla-Evangelista *et al.*, 2013). Indeed, pennycress is a member of the Brassicaceae family and is adapted to a wide range of climate conditions (Vaughn *et al.*, 2005; Cermak *et al.*, 2013; Hojilla-Evangelista *et al.*, 2013). It germinates in the autumn and grows slowly during the winter months. Following the flowering period in the spring, pennycress seeds can be harvested before summer crops are planted (Fan *et al.*, 2013). Thus, pennycress is capable of growing in a rotation with commodity crops without displacing them (Isbell, 2009; Phippen and Phippen, 2012; Cermak *et al.*, 2013). The potential average yield of pennycress seeds is 1500kg ha^–1^, which is equivalent to 600–1200 l ha^–1^ of oil in comparison with 450 and 420–640 l ha^–1^ in the cases of soybean and camelina oils, respectively (Boateng *et al.*, 2010; Phippen and Phippen, 2012). Therefore, pennycress has been studied as an alternative crop that can be used for biofuel. Harvested pennycress seeds contain ~36% oil of which ~94% are unsaturated fatty acids that confer specific physico-chemical properties to pennycress oil. The most abundant unsaturated fatty acid is erucic acid [(Z)-docos-13-enoic acid], a monounsaturated fatty acid with 22 carbons. Pennycress oil has been shown to be suitable for biodiesel production due to its high cetane number of 59.8 and excellent low temperature properties (Moser *et al.*, 2009*a*). These characteristics meet the US biodiesel standard ASTM D6751. Furthermore, results from a life cycle assessment revealed that renewable fuels produced from pennycress oil, in combination with hydrogenation, deoxygenation, isomerization, and hydrocracking reactions, could qualify as a biomass-derived diesel according to the Renewable Fuels Standard (RFS2) (Fan *et al.*, 2013). Therefore, further increases in oil accumulation and erucic acid level by breeding and/or metabolic engineering will ensure pennycress economical viability as a dedicated bioenergy crop. Understanding the biochemical pathways involved in oil synthesis in pennycress is hence timely to guide future crop improvement efforts.

In plants, different pathways in central metabolism are responsible for allocating the carbon skeletons, reducing power and energy required for fatty acid synthesis. Underlying the pathways that are actively involved in erucic acid synthesis in pennycress requires a relatively new discipline known as metabolomics (Cocuron *et al.*, 2014). As an alternative to genomics, transcriptomics, and proteomics, metabolomics plays a pivotal role in investigating genotype–phenotype relationships by quantitative profiling of metabolites in a given organism (Ogura *et al.*, 2013). The strength of metabolomics lies in the fact that chemical compounds serve as a direct signature of biochemical activity, unlike genes and proteins which are prone to a variety of modifications. As of today, two major approaches have been commonly used in metabolomics; untargeted and targeted (Patti *et al.*, 2012). The untargeted approach, known as metabolite fingerprinting, involves the profiling of all compounds present, whereas the targeted approach refers to the quantitative measurement of specific intermediates within given metabolic pathways (Ogura *et al.*, 2013). On the one hand, metabolite fingerprinting can be conducted with gas chromatography–mass spectrometry (GC-MS) (Fiehn, 2008) and/or liquid chromatography–mass spectrometry (LC-MS) which are powerful analytical techniques to unravel the metabolic state of a given organism. On the other hand, obtaining quantitative information on metabolites involved in core biochemical pathways becomes possible with an approach of targeted metabolomics. However, a challenge in accomplishing such a task relies on the choice of the instruments that are capable of detecting and quantifying low concentrations of intermediates that are of interest. Among all the instruments commonly used in metabolomics, liquid chromatography–tandem mass spectrometry (LC-MS/MS) has been given special emphasis due to its high accuracy and sensitivity (Bajad *et al.*, 2006; Luo *et al.*, 2007; Cocuron *et al.*, 2014). LC-MS/MS combines two main modules: liquid chromatography and mass spectrometry. In liquid chromatography, a column separates metabolites according to their chemical properties. Afterwards, these separated compounds undergo electrospray ionization (ESI), producing specific parent/daughter ions that are in turn detected by a triple-quadrupole mass spectrometer in multiple reaction monitoring (MRM) mode (Luo *et al.*, 2007; Cocuron *et al.*, 2014). In previous studies, LC-MS/MS has been shown to be a powerful tool for separating and quantifying known intermediates of central metabolic pathways including glycolysis, the oxidative pentose phosphate pathway (OPPP), and the tricarboxylic acid (TCA) cycle (Koubaa *et al.*, 2013; Cocuron and Alonso, 2014; Cocuron *et al.*, 2014). Therefore, targeted metabolomics studies should highlight which pathways are metabolically active during fatty acid synthesis through the quantification of signature metabolites using LC-MS/MS.

In this work, both qualitative and quantitative approaches were combined to understand the biochemical basis of oil synthesis in pennycress embryos by: (i) analysing the biomass accumulation that determined the main carbon sinks; (ii) conducting a metabolomic profiling study using GC-MS to identify the main classes of metabolites present in pennycress embryos; and (iii) quantifying intracellular compounds involved in central metabolism through LC-MS/MS.

## Materials and methods

### Chemicals

Metabolite standards, 3 N methanolic HCl, and toluene were purchased from Sigma. [U-^13^C]Glucose, [U-^13^C]glycine, and [U-^13^C]fumarate were obtained from Isotec. Potassium hydroxide (KOH), methylene chloride, ethoxyamine hydrochloride, and MSTFA+1% TMCS (*N*-methyl-*N*-trimethylsilytrifluoroacetamide plus 1% trimethylchlorosilane), solvents for GC-MS and LC-MS/MS, were purchased from Fisher Scientific. Gibberellins (GA4/GA7) and Murashige and Skoog basal salt were ordered from PhytoTechnology Laboratories.

### Plant growth

Pennycress seeds of the Ames 30982 accession were obtained from the North Central Regional Plant Introduction Station. The seeds were germinated on plates prior to transfer to pots (Supplementary Fig. S1 at *JXB* online). Briefly, the seeds were sterilized for 5min with 50% bleach in a 2ml tube and rinsed with sterile water a total of four times. Then, the seeds were placed between two aseptic Whatman papers in a 100×15mm glass Petri dish. Sterile Murashige and Skoog salt medium containing 1mM G4/G7 gibberellins, pH 6.0, was added and the plate was sealed with parafilm. Seeds were allowed to germinate for 3–5 d at 22 °C. Finally, the germinated kernels were transferred to pots (14 cm×14 cm×18cm), and grown in a growth chamber at 22 °C under a constant light intensity of 200 μmol m^–2^ s^–1^ and a 16h/8h day/night cycle. Upon emergence of the first pair of true leaves, the plants were transferred to a cold room (4 °C) for 3 weeks. The light intensity and day/night cycle were 100 μmol m^–2^ s^–1^ and 10h/14h, respectively. This step was crucial in ensuring that plants flowered later on. The plants were then placed back into their initial growth chamber and allowed to grow until maturity. The pennycress flowers were hand pollinated and tagged every day in order to study the embryo metabolism at different developmental stages.

### Biomass extraction

Oil, proteins, and starch were sequentially extracted as previously described (Cocuron *et al.*, 2014). A 1:5 dilution was applied to the fatty acid methyl ester (FAME) samples. The remaining pellet after oil, protein, and starch extraction was considered to represent the cell wall.

### Biomass quantification

#### Oil

Oil content was determined by GC-MS. FAMEs were analysed using a Thermo Trace 1310 gas chromatograph coupled to an ISQ single quadrupole mass spectrometer. FAME derivatives were separated using an Omegawax 250 capillary (30 m×0.25 mm×0.25 μm) column from Supelco at a constant flow rate of 1.4ml min^–1^. Helium was used as the carrier gas. The GC conditions were as follows: initial temperature was set to 170 °C and held for 30 s. The oven temperature was then raised to 245 °C at 100 °C min^–1^ and held for 8.75min. The injection temperature was fixed at 225 °C and the injection mode set to split with a split ratio of 10. For the MS analysis, the mass spectra were acquired using electron impact (EI) ionization in positive ion mode. The ion source and the interface temperatures were set to 200 °C and 250 °C, respectively. GC-MS data were acquired and processed using Xcalibur software. FAME derivatives were identified using the NIST 11 library and neat FAME standards purchased from Sigma.

#### Proteins, starch, and cell wall

Proteins and starch were quantified following the steps previously described (Cocuron *et al.*, 2014). Cell wall was estimated by subtracting oil, protein, and starch content from the total dry weight (DW).

### Metabolite extraction

Metabolites were extracted from pennycress embryos at six different stages [11, 13, 15, 17, 19, and 21 days after pollination (DAP)] using boiling water, as previously described (Cocuron *et al.*, 2014). Prior to extraction, 500, 500, and 1000 nmol of [U-^13^C]glucose, [U-^13^C]glycine, and [U-^13^C]fumarate were added, respectively, as internal standards. The hot water extraction was used for the untargeted and targeted metabolomics studies.

### GC-MS analysis of intracellular metabolites

#### Derivatization

Extracted and lyophilized metabolites were derivatized as previously described (Koek *et al.*, 2006) with minor modifications. Briefly, 200 μl of methylene chloride was added and the samples were dried under a stream of nitrogen. This step was repeated twice. Then, 100 μl of pyridine was added to the vials along with 50 μl of a 56mg ml^**–1**^ ethoxyamine hydrochloride solution in pyridine. Samples were flushed with nitrogen for 10 s, resuspended using a vortex, and incubated at 40 °C for 90min in a dry bath. Finally, 350 μl of MSTFA+1% TMCS reagent was added to the samples which were flushed with nitrogen for 10 s and incubated at 40 °C for 50min.

#### GC-MS analysis

Alkylsilyl derivates were analysed using a Thermo Trace 1310 gas chromatograph coupled to an ISQ single quadrupole mass spectrometer. Alkylsilyl derivates were separated using a TG-5MS capillary (30 m×0.25 mm×0.50 μm) column from Thermo Scientific at a constant flow rate of 1.4ml min^–1^. Helium was used as the carrier gas. The GC conditions were as follows: initial temperature was set to 70 °C and held for 5min. The oven temperature was then raised to 235 °C at 3 °C min^–1^. A second ramp was applied at a rate of 6 °C min^–1^ to reach a final temperature of 320 °C which was held for 5min. The injection temperature was fixed at 240 °C and the injection mode was set to split with a split ratio of 3.6. For the MS analysis, the mass spectra were acquired using EI ionization in positive ion mode. The ion source and the interface temperatures were set to 300 °C and 325 °C, respectively. GC-MS data were acquired and processed using Xcalibur software. Alkylsilyl derivatives were identified using the NIST 11 library.

### LC-MS/MS quantification of intracellular metabolites

After lyophilization, extracts were resuspended in 500 μl of nanopure water and vortexed. A 200 μl aliquot of sample was loaded onto a 0.2 μm nanosep MF centrifugal device in order to quantify the sugars. The remaining 300 μl was transferred to a 3kDa Amicon Ultra 0.5ml filtering device for the quantification of amino acids, phosphorylated compounds, and organic acids. The samples were spun at 14 000 *g* for 45min at 4 °C. The intracellular metabolites were separated and quantified as previously described (Cocuron *et al.*, 2014) with minor modifications.

#### Sugars and sugar alcohols

A 15 μl aliquot of extract was diluted in a LC-MS/MS vial containing 975 μl of acetonitrile/water (60:40) solution, and 10 μl of the diluted sample was injected onto the LC-MS/MS column.

#### Amino acids

A 20 μl aliquot of extract was added to a vial containing 880 μl of nano-pure water and 100 μl of 10mM hydrochloric acid, and 10 μl of the diluted sample was injected onto the column.

#### Phosphorylated compounds and organic acids

A 20 μl aliquot of sample was diluted in 180 μl nanopure water, and 20 μl was injected onto the column.

### Statistical analyses

Two-tailed, type 3 Student’s tests (*t*-test) were performed considering as statistically significant *P*-values <0.05. Clustering analyses were performed using MetaboAnalyst v2.5 (Xia *et al.*, 2009, 2012), a free online software (www.metaboanalyst.ca). Briefly, for each metabolite, the quantities across different developmental stages were divided by the highest one. Then, the relative values were uploaded in MetaboAnalyst using the format of samples in row (unpaired). Finally, the K-means partitional clustering was performed by the software.

## Results

### Biomass accumulation in developing pennycress embryos

**Fig. 1.**
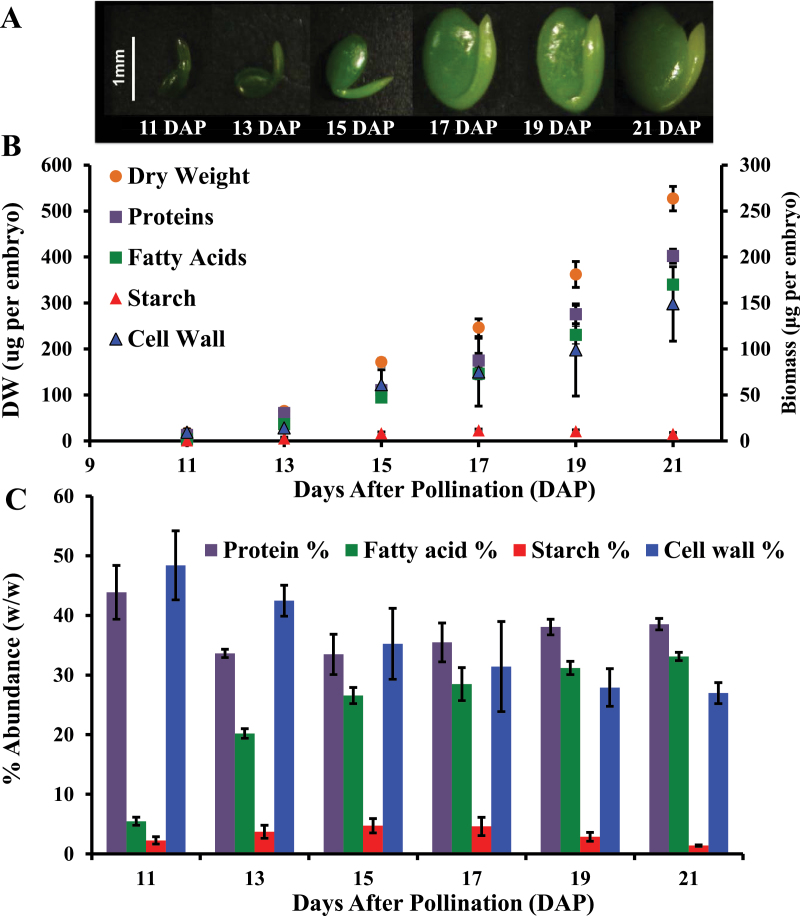
Biomass composition of pennycress embryos at different stages of development. (A) Pictures of the embryos at different stages of development under a dissecting microscope. (B) Biomass accumulation rate of pennycress embryos. The orange circles, purple squares, green squares, red triangles, and blue triangles, respectively, represent the dry weight, the amounts of protein, fatty acid, starch, and cell wall accumulating in a pennycress embryo (*n*=4 biological replicates). (C) Biomass abundance in pennycress embryo. The purple, green, red, and blue bars are associated, respectively, with the percentage (w/w) of protein, fatty acid, starch, and cell wall characterizing a single embryo. Error bars are the SD of four biological replicates.

Biomass components are the final products of central metabolism, and their relative abundance reflects the allocation of carbon by primary metabolic pathways. In order to characterize the main carbon sinks and their accumulation rates, pennycress embryos were dissected at different stages (Fig. 1A) and then dried prior to biomass sequential extraction (Cocuron *et al.*, 2014). Fatty acids, proteins, starch, and cell wall were quantified as described in the Materials and methods. A pennycress embryo grew on average 50.2 μg (DW d^–1^ (*R* ^2^=0.97), accumulating fatty acids, protein, cell wall, and starch with the rates of 16.8 (*R* ^2^=0.94), 19.3 (*R* ^2^=0.95), 12.8 (*R* ^2^=0.95), and 1.9 μg d^–1^ (*R* ^2^=0.97), respectively (Fig. 1B). The protein:fatty acid ratio in pennycress embryos dropped from 8.0 at 11 DAP to 1.2 at 21 DAP, indicating an increase in oil accumulation (Fig. 1C). Fatty acid composition varied across developmental stages to reach a steady state at 15 DAP. Indeed, linoleic acid (C18:2) was found to be the most abundant at 11 DAP (33.8±1.5) whereas erucic acid (C22:1) was under the limit of detection. Then, at 19 DAP, erucic acid became the most abundant fatty acid, reaching a plateau at 36% (Supplementary Fig. S2 at *JXB* online).

### Metabolite profiling in pennycress embryos

**Fig. 2.**
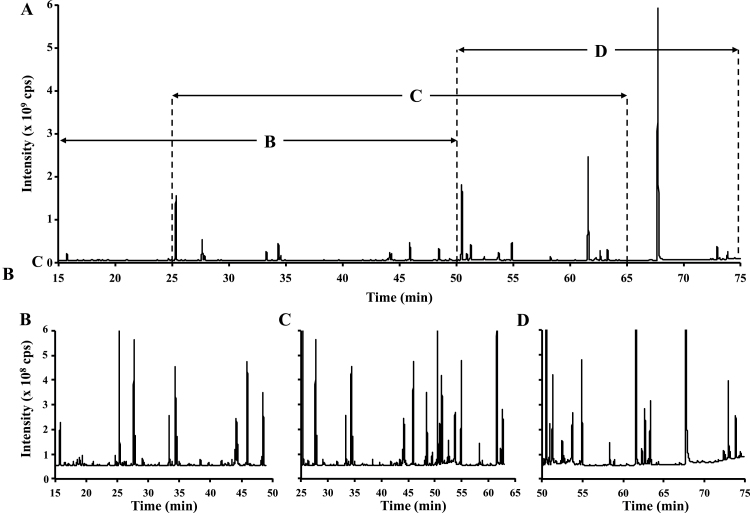
Metabolite profiling of pennycress embryos at 17 DAP. (A) GC-MS chromatogram of 17 DAP pennycress embryos obtained after MSTFA derivatization. Enlarged chromatogram areas depicting the main classes of compounds, (B) amino acids, (C) organic acids, and (D) sugars found in pennycress embryos. The NIST 11 library was used to assign the different peaks.

During the developmental process, embryos produce a wide variety of metabolites in a temporal fashion as a result of changes in their metabolism. Metabolite profiling, also known as untargeted metabolomics, enables the detection of intracellular compounds at a given time. Through this approach one can gain qualitative (rather than quantitative) information about specific classes of intermediates accumulating at the same time as the synthesis of a product of interest. In this study, metabolite profiling was used to characterize all the compounds that were present during fatty acid synthesis. For this purpose, intracellular metabolites were extracted from 17 DAP pennycress embryos with cold methanol:chloroform:water (MCW 2.5:1:1, v:v:v) (Fiehn, 2006) or boiling water (Alonso *et al.*, 2010*b*) and then were chemically modified with MSTFA+1% TMCS (Koek *et al.*, 2006). Through the comparison between GC-MS profiles of the derivatized metabolites, boiling water was shown to be the most suitable method, enabling the detection of 385 peaks versus 344 for MCW (data not shown). A total of 112 peaks out of 385 were assigned with a probability ≥50% using the NIST 11 library (Fig. 2; Supplementary Table S1 at *JXB* online). The identification of the detected peaks qualitatively showed the presence of three main classes of metabolites (sugars, amino acids, and organic acids), and to a lesser extent, alkaloids, polyamines, phosphorylated metabolites, and free fatty acids (Fig. 2; Supplementary Table S1).

### Comparative metabolomics analyses of developing pennycress embryos

**Fig. 3.**
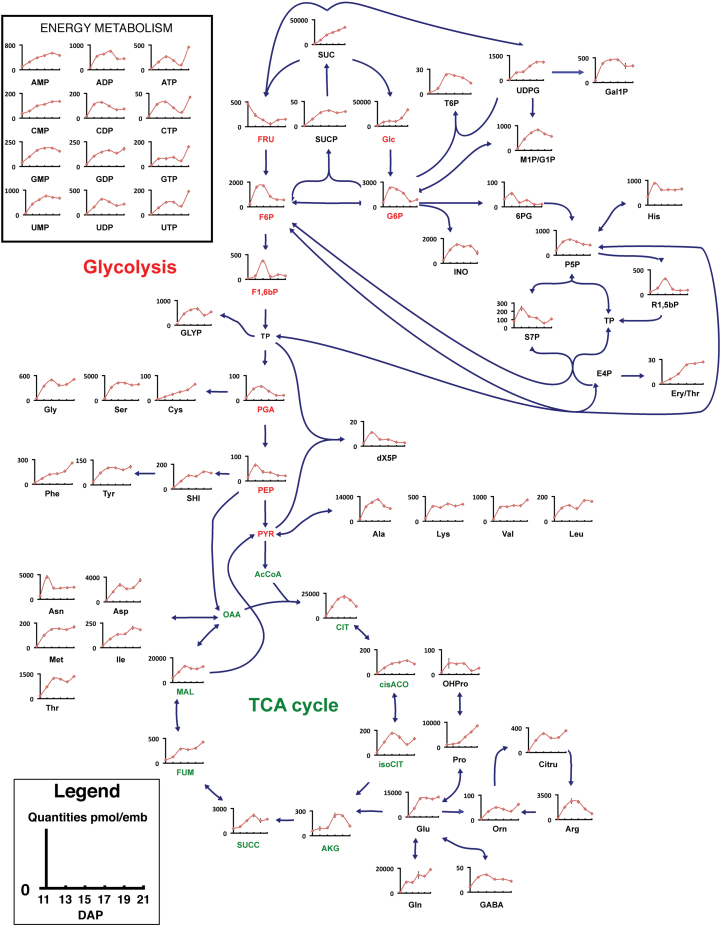
Metabolic map of pennycress embryos at different stages of development. Values are expressed in pmol per embryo and are the average ±SD of three biological replicates from embryos harvested at 11, 13, 15, 17, 19, and 21 DAP. SUC, sucrose; FRU, fructose; GLC, glucose; INO, inositol; GLY, glycerol; Ery/Thr, erythritol/threitol; Ala, alanine; Arg, arginine; Asn, asparagine; Asp, aspartate; Cys, cysteine; Lys, lysine; Gln, glutamine; Glu, glutamate; Gly, glycine; His, histidine; OHPro, hydroxyproline; Leu, leucine; Ile, isoleucine; Met, methionine; Phe, phenylalanine; Pro, proline; Ser, serine; Thr, threonine; Tyr, tyrosine; Val, valine; GABA, 4-aminobutyric acid; Orn, ornithine; Citru, citrulline; T6P, trehalose 6-phosphate; UDPG, UDP-glucose; SUCP, sucrose 6-phosphate; G1P, glucose 1-phosphate; M1P/G1P, mannose 1-phosphate/glucose 1-phosphate; F6P, fructose 6-phosphate; G6P, glucose 6-phosphate; 6PG, 6-phosphogluconic acid; P5P, pentose 5-phosphate; R1,5-bP, ribulose 1,5-bisphosphate; S7P, sedoheptulose 7-phosphate; E4P, eryhtrose 4-phosphate; F1,6bP, fructose 1,6-bisphosphate; GLYP, glycerol-phosphates; TP, triose phosphates; PGA, 2–3 phosphoglycerates; dX5P, deoxyxylulose 5-phosphate; PEP, phosphoenolpyruvate; SHI, shikimate; PYR, pyruvate; AcCoA, acetyl-CoA; CIT, citrate; cisACO, cis-aconitate; isoCIT, isocitrate; AKG, α-ketoglutarate; SUCC, succinate; FUM, fumarate; MAL, malate; OAA, oxaloacetate. Metabolites coloured in red and green correspond to glycolysis and the TCA cycle, respectively.

For the purpose of quantifying the compounds characterized by metabolite profiling, boiling water extraction was performed on pennycress embryos harvested at different stages of development. Extracted compounds were then analysed by LC-MS/MS and quantified according to ^13^C-labelled internal standards as well as standard curves generated for each metabolite. The percentage recovery of this method was previously determined in plant tissues for each metabolite (Cocuron *et al.*, 2014). Intermediates from glycolysis, the OPPP, the TCA cycle, and the Calvin cycle were measured by LC-MS/MS, indicating that all these pathways are active in developing pennycress embryos (Fig. 3; Supplementary Table S2 at *JXB* online).

Sugars are the principal source of carbon provided by the mother plant to the embryos (Schwender and Ohlrogge, 2002; Sriram *et al.*, 2004; Goffman *et al.*, 2005; Alonso *et al.*, 2007, 2010*a*; Allen *et al.*, 2009; Lonien and Schwender, 2009). Sucrose and glucose were quantified as the main free sugars in developing pennycress embryos. Their levels increased by 33-fold, from 1033.2±54.3 pmol to 33479.1±1031.6 pmol per embryo for glucose, and from 1161.7±130.0 pmol to 34963.7±112.4 pmol per embryo for sucrose (Fig. 3; Supplementary Table S2 at *JXB* online). The main sugar alcohols were found to be sorbitol and inositol, with respective levels of 949.1±281.5 pmol and 844.8±155.5 pmol per embryo at 21 DAP (Fig. 3; Supplementary Table S2).

Plant embryos not only receive free amino acids as the source of nitrogen but also produce their own for protein biosynthesis (Schwender and Ohlrogge, 2002; Goffman *et al.*, 2005; Alonso *et al.*, 2007, 2010a; Allen *et al.*, 2009; Lonien and Schwender, 2009). The total amino acid content underwent a 10-fold increase in pennycress embryos between 11 and 21 DAP. Alanine, asparagine, aspartate, glutamate, glutamine, proline, and serine were the seven most abundant amino acids across different developmental stages. Indeed, they represented between 83% and 90% of the total amino acids (Fig. 3; Supplementary Table S2 at *JXB* online). Besides serine that is synthesized from 3-phosphoglycerate, the six others are all produced from organic acids, at the level of the TCA cycle (Fig. 3). The TCA cycle is also important for generating reducing power (FADH_2_ and NADH) that can be used for biomass synthesis and/or for ATP production by oxidative phosphorylation. Malate and citrate, which are involved in fatty acid synthesis and elongation, respectively (Fatland *et al.*, 2000; Nikolau *et al.*, 2000; Alonso *et al.*, 2010*a*; Baud and Lepiniec, 2010), reached 94% of the total organic acids at 13 DAP (Fig. 3; Supplementary Table S2).

Phosphorylated metabolites are key intermediates of glycolysis, OPPP, and the Calvin cycle. Therefore, measuring these compounds is essential to assess central metabolism. The major phosphorylated compounds were found to be glucose 6-phosphate, fructose 6-phosphate, and pentose 5-phosphates at 11 DAP, with respective levels of 536.4±30.5, 231.6±44.7, and 206.1±22.6 pmol per embryo, and remained high during the development of the embryo (Fig. 3; Supplementary Table S2 at *JXB* online). At 21 DAP, UDP-glucose became the most abundant phosphorylated metabolite (1112.5±66.5 pmol per embryo) along with glucose 1-phosphate/mannose 1-phosphate (569.3±30.1 pmol per embryo); these are major precursors for cell wall biosynthesis. Glycerol phosphate levels increased by 7-fold between 11 and 21 DAP; this metabolite provides the glycerol part of the triacylglycerols.

**Fig. 4.**
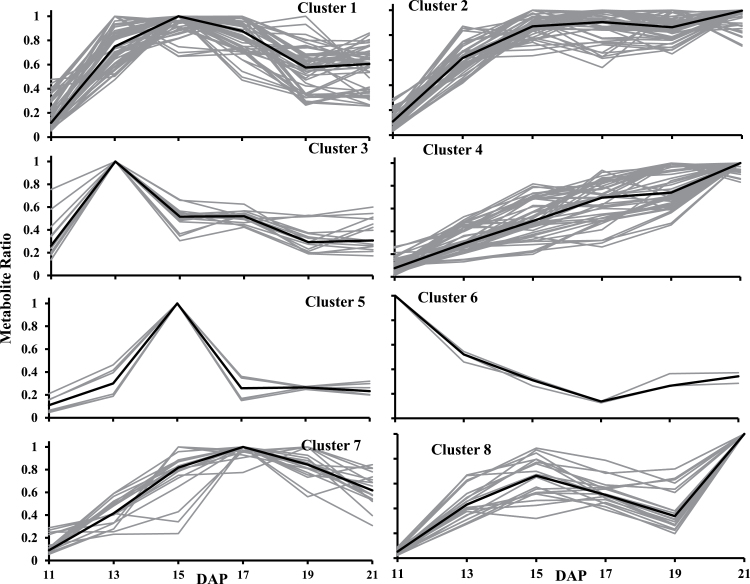
Metabolite clustering of pennycress embryos across different developmental stages. Metabolites were clustered using MetaboAnalyst v2.5. The black lines represent median intensities of corresponding clusters that were obtained from K-means analysis.

In order to group metabolites that share a similar pattern of accumulation over different stages of development, a partitional clustering analysis was performed using MetaboAnalyst (Xia *et al.*, 2009, 2012). Intracellular metabolites in developing pennycress embryos were found to gather in eight clusters (Fig. 4; Table 1). The majority of compounds in cluster 1 increased from 11 to 15 DAP and then decreased; 33% of the phosphorylated metabolites grouped into this cluster. The intermediates from cluster 2, among which nine were amino acids, rapidly increased to reach a plateau at 15 DAP. Clusters 3, 5, and 7 peaked at 13, 15, and 17 DAP, respectively, and then decreased. Interestingly, fructose 1,6-bisphosphate and ribulose 1,5-bisphosphate grouped in cluster 7, and four of the organic acids grouped in cluster 5. Metabolites gradually accumulating during embryos development were gathered in cluster 4; the main sugars (glucose and sucrose), several of the most abundant amino acids (glutamine and proline), and phosphorylated compounds (UDP-glucose) were found in this cluster. Fructose was the only metabolite steadily decreasing over time, and therefore was separated from all the other intermediates (cluster 6). Finally, all the nucleotide triphosphates grouped together in cluster 8; these had a peak at 15 DAP and then reached an optimum at 21 DAP.

**Table 1.** Clusters of metabolites in developing pennycress embryos Metabolites were clustered using MetaboAnalyst v2.5.

Cluster	Metabolites			
	Sugars and sugar alcohols	Amino acids	Phosphorylated compounds	Organic acids
1	INO	Arg, Ala, His, GABA, OHPro	PGA, F6P, G6P, ADP, IMP, CDP, Gal1P, GLYP, UDP, P5P	isoCIT
2		Glu, Gly, Lys, Met, Ser, Thr, Tyr, Val	GDP, GMP, Man6P, UMP, SUCP	MAL, CIT
3		Asn	6PG, dX5P, S7P, PEP	
4	Glc, Sorb, Ery/Thr, pentitols, SUC	Cys, Phe, Pro, Gln, Ile	UDPG, CMP, AMP,	transACO, FUM, SHI
5			F1,6bP, R1,5bP	
6	FRU			
7			T6P, G1P	AKG, cisACO, CIT, SUCC
8		Asp, Orn	ATP, CTP, GTP, UTP	

For abbreviations, see the legend of Fig. 3.

Abbreviations: DAP, days after pollination; DW, dry weight; FAMEs, fatty acid methyl esters; MCW, methanol:chloroform:water; MRM, multiple reaction monitoring; MSTFA, *N*-methyl-*N*-trimethylsilytrifluoroacetamide; TMCS, trimethylchlorosilane; OPPP, oxidative pentose-phosphate pathway; TCA, tricarboxylic acid.

## Discussion

Pennycress naturally accumulates high levels of erucic acid in its embryos, which makes it a promising biodiesel and industrial crop (Moser *et al*., 2009*a*, *b*). Understanding the biochemical basis of oil synthesis in pennycress embryos is therefore relevant to guide future breeding and/or metabolic engineering efforts. In plants, fatty acid synthesis occurs predominantly in plastids and requires carbon (acetyl-CoA), energy (ATP), and reducing power (NADH and NADPH), which are provided *in situ* by the activity of central metabolism (Hills, 2004; Baud and Lepiniec, 2010). The main carbon sinks in pennycress embryos were found to be proteins, fatty acids, and cell wall, which represented 38.5, 33.2, and 27.0%, respectively of the biomass at 21 DAP (Fig. 1). In comparison, the embryos of other Brassicaceae, such as *Arabidopsis thaliana* and *Physaria fendleri*, accumulate up to 40% and 55% (w/w) of oil (Lonien and Schwender, 2009; Cocuron *et al.*, 2014); these levels could be potentially achieved in pennycress too. Erucic acid reached its highest level of ~36% of the total fatty acids in pennycress embryos at 19 DAP (Supplementary Fig. S2 at *JXB* online). According to the results above, future crop improvement might involve the increase of: (i) the carbon flow towards oil synthesis; and (ii) the elongation of oleic acid (C18:1) to erucic acid (C22:1). Indeed, recent genetic manipulations successfully enhanced the percentage of erucic acid in crambe (Li *et al.*, 2012), and would be a promising approach for pennycress.

Metabolomics emerged as a powerful tool to assess the metabolic state of a given organism/tissue (Patti *et al.*, 2012), specifically quantifying key intermediate compounds involved in primary pathways (Huck *et al.*, 2003; Bajad *et al.*, 2006; Luo *et al.*, 2007; Alonso *et al.*, 2010*b*; Koubaa *et al.*, 2013; Cocuron and Alonso, 2014; Cocuron *et al.*, 2014). In this study, intracellular metabolites were extracted from pennycress embryos using boiling water, which has been shown to be the most efficient method to extract water-soluble compounds from various biological sources (microorganisms, mammalian cells, plant tissues, etc.) with the maximum recovery (Alonso *et al.*, 2010*a*; El Rammouz *et al.*, 2010; Cocuron *et al.*, 2014). Two metabolomic approaches were applied in this work to fingerprint the physiological activities of pennycress embryos. The first one, untargeted metabolomics, is a purely qualitative method: it has been widely used to assess the global metabolite profile of a sample and to detect novel entities (Koek *et al.*, 2006; Patti *et al.*, 2012; Wolfender *et al.*, 2013; Macel *et al.*, 2014; Mie *et al.*, 2014). In this study, metabolite fingerprinting and the main classes of intermediates in pennycress embryos were determined using GC-MS and a structural database (Fig. 2). The second approach is more targeted and relies on the selective quantification of key intracellular metabolites. The levels of intermediates may be expressed as relative (Rolletschek *et al.*, 2011; Borisjuk *et al.*, 2013) or absolute values (Urakami *et al.*, 2010; Cocuron *et al.*, 2014; Wu *et al.*, 2014); with absolute quantities offering the possibility to draw comparisons between different metabolites, in various tissues, conditions, etc. High-throughput LC-MS/MS methods have been recently developed and validated to separate and quantify the intermediates and precursors for plant biomass synthesis: amino acids, sugars/sugar alcohols, phosphorylated compounds, and organic acids, which represent ~100 metabolites (Alonso *et al.*, 2010*b*; Koubaa *et al.*, 2013; Cocuron and Alonso, 2014; Cocuron *et al.*, 2014). These methods have been applied here to compare intracellular metabolite levels in pennycress embryos at different stages of development (Fig. 3; Supplementary Table S2 at *JXB* online). It is important to note that the targeted metabolomics study presented here was performed with the future objective to carry out flux analysis. In the plant field, it is the norm to describe units on a per embryo basis for flux analysis, and in the future it will be useful in comparing the net accumulation of intermediate metabolites with the carbon fluxes through the metabolic pathways. However, metabolite quantities have also been reported here per mg DW (Supplementary Table S3) in order to facilitate the comparison with other plants, organs, etc.

In this study, metabolomics was used to probe the activity of central metabolic pathways and to determine their implication in fatty acid synthesis. First, the main intracellular sugars and amino acids were found to be sucrose, glucose, and glutamine (Fig. 3; Supplementary Table S2 at *JXB* online), which were grouped into cluster 4 (Fig. 4; Table 1); these are respectively common sources of carbon and nitrogen for developing plant embryos (Schwender and Ohlrogge, 2002; Sriram *et al.*, 2004; Goffman *et al.*, 2005; Alonso *et al.*, 2007, 2010*a*; Allen *et al.*, 2009; Lonien and Schwender, 2009). In developing pennycress embryos, fructose was the only metabolite in cluster 6 (Fig. 4; Table 1), with its level decreasing over time. This observation, together with the high levels of sucrose and glucose, indicate that sucrose might be stored rather than cleaved into hexoses via the invertase. In agreement with this study, targeted metabolomics on the embryos of another Brassicaceae, *Physaria fendleri*, also reported the accumulation of sucrose across developmental stages (Cocuron *et al.*, 2014). A variety of integrative functions have been suggested for sucrose storage in plant embryos, including modulation of gene expression, protein turnover, and a trigger to induce storage pathway (Weber *et al.*, 1997; Farrar *et al.*, 2000; Borek and Nuc, 2011). In earlier studies, storage activity was shown to occur in both avocado and field bean embryos when the sucrose level increased (Weber *et al.*, 1997; Sanchez-Romero *et al.*, 2002). Additionally, sucrose storage is involved in the acquisition of desiccation tolerance during seed development and maturation (Businge *et al.*, 2013). Sugars received by the embryos are metabolized in the cytosol, supplying carbon skeletons to biomass synthesis (Hills, 2004; Baud and Lepiniec, 2010). Secondly, the main organic acids were malate and citrate (Fig. 3; Supplementary Table S2 at *JXB* online) which have been shown to provide acetyl-CoA for fatty acid synthesis and elongation, respectively (Fatland *et al.*, 2000; Nikolau *et al.*, 2000; Alonso *et al.*, 2010*a*; Baud and Lepiniec, 2010). Thirdly, the presence of ribulose 1,5-bisphosphate (Fig. 3; Supplementary Table S2), a metabolite specific to the Calvin cycle, and the green colour of the embryos (Fig. 1) indicate that they are photosynthetically active between 11 and 21 DPA. It has been observed that in green seeds, light energy can be used by chloroplasts to generate ATP and NADPH (Browse and Slack, 1985; Ohlrogge *et al.*, 2004; Schwender *et al*., 2004, 2006; Goffman *et al.*, 2005). Therefore, photosynthesis might provide part of the energy and reductant necessary for fatty acid production in developing pennycress embryos. Furthermore, ribulose 1,5-bisphosphate is the substrate of the ribulose 1,5-bisphosphate carboxylase/oxygenase (RuBisCo). High activities of this enzyme have been measured in Brassicaceae embryos (King *et al.*, 1998; Ruuska *et al.*, 2004) where it has been shown to fix the CO_2_ released by the pyruvate dehydrogenase, increasing the efficiency of carbon use (Schwender *et al.*, 2004). Interestingly ribulose 1,5-bisphosphate uniquely clustered with fructose 1,6-bisphosphate (Fig. 4; Table 1), which indicates that a large portion of the fructose 1,6-bisphosphate might be produced by the Calvin cycle. Finally mitochondrial respiration and the OPPP usually are the two other pathways generating ATP and NADPH, respectively. The level of free amino acids was found to be higher than that of their direct precursors from glycolysis, the TCA cycle, and the OPPP (Fig. 3; Supplementary Table S2), revealing a high flow of carbon through these pathways. Besides photosynthesis, oxidative phosphorylation and the OPPP might be a significant source of energy and reductant for oil synthesis in developing pennycress embryos.

To date, there is only one other quantitative metabolomics study which was conducted on developing embryos of *Physaria fendleri* (Cocuron *et al.*, 2014). The major developmental difference between *Physaria* and pennycress embryos was the rate of DW accumulation being twice faster in pennycress. The comparison of the intracellular compound levels between same stage embryos (i.e. 17 and 27 DAP for pennycress and *Physaria*, respectively) highlighted major biochemical and metabolic differences. First, *Physaria* embryos synthesized more oil (55% versus 33%; w/w). Secondly, besides sucrose, the levels of the other major free sugars were higher whereas hexose-phosphates were lower in *Physaria*, suggesting a faster glycolytic flow in pennycress embryos. Thirdly, the amounts of all the organic acids were lower in *Physaria* by a factor 6–55, which may indicate a slower TCA cycle. Finally, the intermediates of the OPPP (6-phosphogluconate, sedoheptulose 7-phosphate, and pentose-phosphates) and the Calvin cycle (ribulose 1,5-bisphosphate) were found to be higher in pennycress embryos, suggesting a larger flow of carbon through these pathways. The metabolomics study hence revealed the occurrence of key pathways involved in oil production in pennycress embryos. However, the relative contribution of each of these pathways to the synthesis of fatty acids (in terms of carbon skeletons, energy, and reductant), and the potential bottlenecks can only be determined by measuring the *in vivo* metabolic fluxes (Alonso *et al.*, 2010*a*; Dieuaide-Noubhani and Alonso, 2014).

## Supplementary data

Supplementary data are available at *JXB* online.

Figure S1. *Thlaspi arvense* L. plant anatomy.

Figure S2. Fatty acid composition in developing pennycress embryos.

Table S1. Untargeted metabolomics analysis of pennycress embryos at 17 DAP.

Table S2. Targeted metabolomics analyses of pennycress embryos at different developmental stages.

Table S3. Metabolite levels in developing pennycress embryos expressed in pmol mg DW^–1^.

Supplementary Data
