# Supplementary material for: Metabolite fingerprinting of pennycress (Thlaspi arvense L.) embryos to assess active pathways during oil synthesis
Source: J Exp Bot. 2015 Feb 22;66(14):4267–77. doi: 10.1093/jxb/erv020 (PMC4493779; doi:10.1093/jxb/erv020)
Supplement: Supplementary Data [file supp_66_14_4267__index.html]

Metabolite fingerprinting of pennycress (Thlaspi arvense L.) embryos to assess active pathways during oil synthesis — Metabolite fingerprinting of pennycress (Thlaspi arvense L.) embryos to assess active pathways during oil synthesis — Supplementary Data 

# Metabolite fingerprinting of pennycress (*Thlaspi arvense* L.) embryos to assess active pathways during oil synthesis

## Supplementary Data

Data files

**Files in this Data Supplement:**

- Supplementary Data - Supplementary Data
