# Supplementary material for: Metabolite fingerprinting of pennycress (Thlaspi arvense L.) embryos to assess active pathways during oil synthesis
Source: J Exp Bot. 2015 Feb 22;66(14):4267–77. doi: 10.1093/jxb/erv020 (PMC4493779; doi:10.1093/jxb/erv020)
Supplement: Supplementary Data [file supp_erv020_jexbot139030_file001.pdf]

**TITLE PAGE:**

**Date of resubmission:** ~~October 24th~~December 11th, 2014
**Number of tables:** 1
**Number of figures:** 4 (of which ~~1~~ 2 in colour in print)
**Total word count:** 7,~~044~~693
**Supplementary figures:** 2
**Supplementary tables:** 3

**TITLE:**

Metabolite fingerprinting of pennycress (*Thlaspi arvense* L.) embryos to assess active pathways during oil synthesis

**RUNNING TITLE:** Metabolite fingerprinting of pennycress embryos

**AUTHORS:**

Enkhtuul Tsogtbaatar[1], Jean-Christophe Cocuron[1,2], Marcos Corchado Sonera[3], Ana Paula Alonso[1,*]

**ADDRESSES:**

[1] The Ohio State University, Department of Molecular Genetics, Columbus, OH 43210, USA

[2] The Ohio State University, Center for Applied Plant Sciences, Columbus, OH 43210, USA

[3] University of Puerto Rico, Mechanical Engineering Department, Mayagüez, 00681-9000, Puerto Rico

[*] To whom correspondence should be addressed. E-mail: alonso.19@osu.edu

**E-MAIL ADDRESSES:**

Enkhtuul Tsogtbaatar: E-mail: tsogtbaatar.1@osu.edu
Jean-Christophe Cocuron: E-mail: cocuron.1@osu.edu
Marcos Corchado Sonera: E-mail: marcos.corchado@upr.edu
Ana Paula Alonso: E-mail: alonso.19@osu.edu; Phone: +1 (614) 688 7404; Fax: +1 (614) 247 8937

## NOVELTY:

Metabolomics was shown to be a powerful tool, both for quantifying intracellular compounds and qualitatively assessing biochemical pathways. This approach underlined metabolic routes involved in the synthesis of biofuel-relevant oils.

## ABSTRACT:

Pennycress (*Thlaspi arvense* L.), a plant naturalized~~ive~~ to~~plant of~~ North America,~~ naturally~~ accumulates high levels of erucic acid in its seeds, which makes it a promising biodiesel and industrial crop. The main carbon sinks in pennycress embryos were found to be proteins, fatty acids, and cell wall, which respectively represented 38.5%, 33.2%, and 27.0% of the biomass at 21 days after pollination. Erucic acid reached a maximum of 36~~3~~% of the total fatty acids. Together these results indicate that total oil and erucic acid contents could be increased to boost the economic competitiveness of this crop. Understanding the biochemical basis of oil synthesis in pennycress embryos is therefore timely and relevant to guide future breeding and/or metabolic engineering efforts. For this purpose, a combination of metabolomics approaches was conducted to assess the active biochemical pathways during oil synthesis. First, gas chromatography-mass spectrometry profiling of intracellular metabolites highlighted three main families of compounds: organic acids, amino acids, and sugars/sugar alcohols. Second, these intermediates were quantified in developing pennycress embryos by liquid chromatography tandem mass spectrometry (LC-MS/MS) in multiple reaction monitoring mode. Finally, partitional clustering analysis grouped the intracellular metabolites that shared a similar pattern of accumulation over time into eight clusters. This study underlined that: i) sucrose might be stored rather than cleaved into hexoses; ii) glucose and glutamine would be the main sources of carbon and nitrogen, respectively; iii) the glycolysis, the oxidative pentose phosphate pathway, the tricarboxylic acid cycle, and the Calvin cycle were active in developing pennycress embryos.

## KEYWORDS:

alternative crop; erucic acid; GC-MS; jet fuel; LC-MS/MS; metabolomics; oilseed; pennycress; plant metabolism; *Thlaspi arvense* L.; triacylglycerols.

## ABBREVIATIONS:

DAP, days after pollination; DW, dry weight; FAMEs, fatty acid methyl esters; MCW, methanol:chloroform:water; MRM, multiple reaction monitoring; MSTFA, N-methyl-N-trimethylsilytrifluoroacetamide; TMCS, trimethylchlorosilane; OPPP, oxidative pentose-phosphate pathway; TCA, tricarboxylic acid.

## INTRODUCTION:

Petroleum is the largest energy source in the United States, accounting for 28 percent of all energy consumed in 2013 (www.eia.gov). The fact that petroleum-based fuels will eventually be depleted requires the development of renewable fuels. Indeed, biofuel production has attracted considerable research attention in both developing and industrialized countries (Baud *et al.*, 2007). In addition to its ability to mitigate the approaching shortage of petroleum, renewable energy has the additional environmental benefit of being a low contributor of greenhouse gases (Kim and Dale, 2005). Biofuel is a renewable fuel that can be produced from plant biomass components such as oil, starch and cell wall. In the USA, the most common biofuels, such as ethanol and biodiesel, are currently produced from corn, soybean, and other high cost commodity crops (Demirbas, 2009; Kim and Dale, 2005; Moser *et al.*, 2009a). In fact, it seems crucial to address that the biofuel industry not use crops with valuable food applications. However, the availability of other suitable bioenergy plants, such as sugarcane, is limited to certain geographies and climates. Taking these challenges into consideration, the biofuel industry is in need of alternative crops that meet the following criteria: i) a favorable biomass composition for biofuel production, ii) the ability to grow in a variety of soils and climates and iii) no-competitions with food crops. The alternative bioenergy crops that have been studied so far include, but are not restricted to, crambe (Li *et al.*, 2012), camelina (Frohlich and Rice, 2005), brassica carinata (Bouaid *et al.*, 2009), miscanthus (Robson *et al.*, 2013) and sugarcane (Hojilla-Evangelista *et al.*, 2013).

Field pennycress (*Thlaspi arvense* L.; Supplementary Figure S1) is ~~,~~ a winter annual that grows widely across temperate regions of~~is widely spread across~~ North America and the southern hemisphere ~~a~~(Warwick *et al.*, 2002).~~,~~ It has been

identified as an oilseed crop that could be a suitable source for biofuel (Hojilla-Evangelista *et al.*, 2013; Vaughn *et al.*, 2005). Indeed, pennycress is a member of the Brassicaceae family and is adapted to a wide range of climate conditions (Cermak *et al.*, 2013; Hojilla-Evangelista *et al.*, 2013; Vaughn *et al.*, 2005). It germinates in the fall and grows slowly during the winter months. Following the flowering period in the spring, pennycress seeds can be~~are~~ harvested before summer crops are planted (Fan *et al.*, 2013). Thus, pennycress is capable of growing in a rotation with commodity crops without displacing them (Cermak *et al.*, 2013; Isbell, 2009; Phippen and Phippen, 2012). The potential average yield of pennycress seeds is 1500kg/ha, which is equivalent to 600-1200L/ha of oil in comparison with 450 and 420-640L/ha in the cases of soybean and camelina oils, respectively (Boateng *et al.*, 2010; Phippen and Phippen, 2012). Therefore, pennycress has been studied as an alternative crop that can be used for biofuel. Harvested pennycress seeds contain about 36~~5~~% oil of which approximately 94% are unsaturated fatty acids that confer specific physico-chemical properties to pennycress oil. The most abundant one ~~30-33%~~ is erucic acid ((Z)-Docos-13-enoic acid), a monounsaturated fatty acid with 22 carbons. ~~This high content of erucic acid and its overall fatty acid profile~~ Pennycress oil has been shown to be suitable for biodiesel production due to its high cetane number of 59.8 and excellent low temperature properties (Moser *et al.*, 2009a). These characteristics meet the United States biodiesel standard ASTM D6751. Furthermore, ~~confer improved cold temperature properties to the oil, making it suitable for biofuel production.~~ results from a life cycle assessment revealed that renewable fuels produced from pennycress oil, in combination with hydrogenation, deoxygenation, isomerization, and hydrocracking reactions could qualify as a biomass-derived diesel according to the Renewable Fuels Standard (RFS2) (Fan *et al.*, 2013). ~~Furthermore, erucic acid is a valuable precursor for many other industrial applications such as plastics, nylon and high temperature lubricants.~~ Therefore, further increases in oil accumulation and erucic acid level by breeding and/or metabolic engineering will insure pennycress economical viability as a dedicated bioenergy crop. Understanding the biochemical pathways involved in oil synthesis in pennycress is hence timely to guide future crop improvement efforts.

In plants, different pathways in central metabolism are responsible for allocating the carbon skeletons, reducing power and energy required for fatty acid synthesis. Underlying the pathways that are actively involved in erucic acid synthesis in pennycress requires a relatively new discipline known as metabolomics (Cocuron *et al.*, 2014). As an alternative to genomics, transcriptomics and proteomics, metabolomics plays a pivotal role in investigating genotype-phenotype relations by quantitative profiling of metabolites in a given organism (Ogura *et al.*, 2013). The strength of metabolomics lies in the fact that chemical compounds serve as a direct signature of biochemical activity, unlike genes and proteins which are prone to a variety of modifications. As of today, two major approaches have been commonly used in metabolomics; untargeted and targeted (Patti *et al.*, 2012). The untargeted approach, known as metabolite fingerprinting, involves the profiling of all present compounds whereas the targeted one refers to the quantitative measurement of specific intermediates within given metabolic pathways (Ogura *et al.*, 2013). On one hand, metabolite fingerprinting can be conducted with Gas Chromatography-Mass Spectrometry (GC-MS) (Fiehn, 2008) and/or Liquid Chromatography-Mass Spectrometry (LC-MS) which are powerful analytical techniques to unravel the metabolic state of a given organism. On the other hand, obtaining quantitative information on metabolites involved in core biochemical pathways becomes possible with an approach of targeted metabolomics. However, a challenge in accomplishing such a task relies on the choice of the instruments that are capable of detecting and quantifying low concentrations of intermediates that are of interest. Among all the instruments commonly used in metabolomics, liquid chromatography-tandem mass spectrometry (LC-MS/MS) has been given special emphasis due to its high accuracy and sensitivity (Bajad *et al.*, 2006; Cocuron *et al.*, 2014; Luo *et al.*, 2007). LC-MS/MS combines two main modules: liquid chromatography and mass spectrometry. In liquid chromatography, a column separates metabolites according to their chemical properties. Afterwards, these separated compounds undergo electrospray ionization (ESI), producing specific parent/daughter ions that are in turn detected by a triple-quadrupole mass spectrometer in multiple reaction monitoring (MRM) mode (Cocuron *et al.*, 2014; Luo *et al.*, 2007). In previous studies, LC-MS/MS has been shown to be a powerful tool for separating and quantifying

known intermediates of central metabolic pathways including glycolysis, the pentose phosphate pathway and the tricarboxylic acid cycle (Cocuron and Alonso, 2014; Cocuron *et al.*, 2014; Koubaa *et al.*, 2013). Therefore, targeted metabolomics studies should highlight which pathways are metabolically active during fatty acid synthesis through the quantification of signature metabolites using LC-MS/MS.

In this work, we combined both qualitative and quantitative approaches to understand the biochemical basis of oil synthesis in pennycress embryos by: i) analyzing the biomass accumulation that determined the main carbon sinks; ii) conducting an metabolomic profiling study using GC-MS to identify the main classes of metabolites present in pennycress embryos; iii) quantifying intracellular compounds involved in central metabolism through LC-MS/MS.

## MATERIALS AND METHODS:

### *Chemicals*

Metabolite standards, 3N methanolic HCl and toluene were purchased from Sigma. [U-$^{13}$C]glucose, [U-$^{13}$C]glycine and [U-$^{13}$C]fumarate were obtained from Isotec. Potassium hydroxide (KOH), methylene chloride, ethoxyamine hydrochloride, MSTFA + 1% TMCS, solvents for GC-MS and LC-MS/MS were purchased from Fisher Scientific. Gibberellins (GA4/GA7) and Murashige and Skoog (MS) basal salt were ordered from PhytoTechnology Laboratories.

### *Plant growth*

Pennycress seeds of the Ames 30982 accession were obtained from North Central Regional Plant Introduction Station. The seeds were germinated on plate prior to transfer to pots (Supplementary Figure S1). Briefly, the seeds were sterilized for 5 minutes with 50 % bleach in a 2 mL tube and rinsed with sterile water for a total of four times. Then, the seeds were placed between two aseptic Whatman papers in a 100 x 15 mm glass petri-dish. Sterile MS salt medium containing 1 mM G4/G7 gibberellines, pH 6.0, was added and the plate was sealed with parafilm. Seeds were allowed to germinate for 3-5 days at 22°C. Finally, the germinated kernels were transferred to pots (14 cm×14 cm×18 cm), and grown in a growth chamber at 22°C under a constant light intensity of 200 µmol m$^{-2}$ s$^{-1}$ and a 16h/8h day/night cycle. Upon emergence of the first pair of

true leaves, the plants were transferred to a cold room (4°C) for 3 weeks. The light intensity and day/night cycle were 100 µmol $m^{-2}$ $s^{-1}$ and 10h/14h respectively. This step was crucial in having plants flowering later on. The plants were then placed back into their initial growth chamber and allowed to grow until maturity. The pennycress flowers were hand pollinated and tagged every day in order to study the embryo metabolism at different developmental stages.

### *Biomass extraction*

Oil, proteins and starch were sequentially extracted as previously described (Cocuron *et al.*, 2014). A 1:5 dilution was applied to the fatty acid methyl ester (FAME) samples. The remainder pellet after oil, protein and starch extraction was considered to represent the cell wall.

### *Biomass quantification*

*Oil*

Oil content was determined by GC-MS. FAMEs were analyzed using a Thermo Trace 1310 gas chromatograph coupled to an ISQ single quadrupole mass spectrometer. FAME derivatives were separated using an Omegawax 250 capillary (30 m x 0.25 mm x 0.25 µm) column from Supelco at a constant flow rate of 1.4 mL $min^{-1}$. Helium was used as the carrier gas. The GC conditions were as follows: initial temperature was set to 170°C and held for 30 seconds. The oven temperature was then raised to 245°C at 100°C $min^{-1}$ and held for 8.75 minutes. The injection temperature was fixed at 225°C and the injection mode set to split with a split ratio of 10. For the MS analysis, the mass spectra were acquired using electron impact (EI) ionization in positive ion mode. The ion source and the interface temperatures were respectively set to 200°C and 250°C. GC-MS data were acquired and processed using Xcalibur software. FAME derivatives were identified using NIST 11 library and neat FAME standards purchased from Sigma.

*Proteins, ~~and~~ starch, and cell wall*

Proteins and starch were quantified following the steps previously described (Cocuron *et al.*, 2014). Cell wall was estimated by subtracting oil, protein, and starch content from the total dry weight.

### *Metabolite extraction*

Metabolites were extracted from pennycress embryos at six different stages (11, 13, 15, 17, 19 and 21 DAP) using boiling water as previously described (Cocuron *et al.*, 2014). Prior to extraction~~Five hundred~~, 500, 500 and 1000 nmol of [U-$^{13}$C]glucose, glycine and fumarate were added, respectively, as internal standards~~ prior extraction~~. The hot water extraction was used for the untargeted and targeted metabolomics studies.

### *GC-MS analysis of intracellular metabolites*

*Derivatization*

Extracted and lyophilized metabolites were derivatized as previously described (Koek *et al.,* 2006) with minor modifications. Briefly, 200 µL of methylene chloride was added and the samples were dried under a stream of nitrogen. This step was repeated twice. Then, 100 µL of pyridine was added to the vials along with 50 µL of a 56 mg mL$^{-1}$ ethoxyamine hydrochloride solution in pyridine. Samples were flushed with nitrogen for 10 seconds, resuspended using a vortex and incubated at 40 °C for 90 minutes in a dry bath. Finally, 350 µL of MSTFA + 1% TMCS reagent was added to the samples which were flushed with nitrogen for 10 seconds and incubated at 40 °C for 50 minutes.

*GC-MS analysis*

Alkylsilyl derivates were analyzed using a Thermo Trace 1310 gas chromatograph coupled to an ISQ single quadrupole mass spectrometer. Alkylsilyl derivates were separated using a TG-5MS capillary (30 m x 0.25 mm x 0.50 µm) column from Thermo Scientific at a constant flow rate of 1.4 mL min$^{-1}$. Helium was used as the carrier gas. The GC conditions were as follow: initial temperature was set to 70°C and hold for 5 minutes. The oven temperature was then raised to 235°C at 3°C min$^{-1}$. A second ramp was applied at a rate of 6°C min$^{-1}$ to reach a final temperature of 320°C which was held for 5 minutes.

The injection temperature was fixed at 240°C and the injection mode was set to split with a split ratio of 3.6. For the MS analysis, the mass spectra were~~as~~ acquired using electron impact (EI) ionization in positive ion mode. The ion source and the interface temperatures were respectively set to 300°C and 325°C. GC-MS data were acquired and processed using Xcalibur software. Alkylsilyl derivatives were identified using the NIST 11 library.

### *LC-MS/MS quantification of intracellular metabolites*

After lyophilization, extracts were resuspended in 500 µL of nanopure water and vortexed. Two hundred µL of sample was loaded onto a 0.2 µm nanosep MF centrifugal device in order to quantify the sugars. The remaining 300 µL was transferred to a 3 kDa Amicon Ultra 0.5 ml filtering device for the quantification of amino acids, phosphorylated compounds and organic acids. The samples were spun at 14,000×*g* for 45 minutes at 4°C. The intracellular metabolites were separated and quantified as previously described (Cocuron *et al.*, 2014) with minor modifications.

#### *Sugars and sugar alcohols*

Twenty five µL of extract was diluted in a LC-MS/MS vial containing 975 µL acetonitrile/water (60:40) solution, and 10 µL of the diluted sample was injected onto the LC-MS/MS column.

#### *Amino acids*

Twenty µL of extract was added to a vial containing 880 µL of nano-pure water and 100 µL of 10 mM hydrochloric acid, and 10 µL of the diluted sample was injected onto the column.

#### *Phosphorylated compounds and organic acids*

Twenty µL of sample was diluted in 180 µL nanopure water, and 20 µL was injected onto the column.

### *Statistical analyses*

Two tailed, type 3 Student's tests (t-test) were performed considering statistically significant p-values below 0.05. Clustering analyses were performed using

MetaboAnalyst v2.5 (Xia *et al.*, 2012; Xia *et al.*, 2009), a free online software (www.metaboanalyst.ca). Briefly, for each metabolite, the quantities across ~~the~~ different developmental stages were divided by the highest one. Then, the relative values were uploaded in MetaboAnalyst using the format of samples in row (unpaired). Finally, the K-means partitional clustering was performed by the software.

## RESULTS:

### *Biomass accumulation in developing pennycress embryos*

Biomass components are the final products of central metabolism and their relative abundance reflects the allocation of carbon by primary metabolic pathways. In order to characterize the main carbon sinks and their accumulation rates, pennycress embryos were dissected at different stages (Fig. 1A) and then dried prior to biomass sequential extraction (Cocuron *et al.*, 2014). Fatty acids, proteins, starch and cell wall were quantified as described in Materials and Methods. A pennycress embryo grew on average 50.2 µg DW day$^{-1}$ ($R^2$ = 0.97), accumulating fatty acids, protein, cell wall and starch with the rates of 16.8 ($R^2$ = 0.94), 19.3 ($R^2$ = 0.95), 12.8 ($R^2$ = 0.95~~7~~) and 1.9 µg day$^{-1}$ ($R^2$ = 0.97), respectively (Fig. 1B). The protein:fatty acid ratio in pennycress embryos dropped from 8.0 at 11 DAP to 1.2 at 21 DAP, indicating an increase in oil accumulation (Fig. 1C). Fatty acid composition varied across ~~the~~ developmental stages to reach a steady state at 15 DAP. Indeed, linoleic acid (C18:2) was found to be the most abundant at 11 DAP (33.8 ± 1.5) whereas erucic acid (C22:1) was under the limit of detection. Then, at 19~~5~~ DAP erucic acid became the most abundant fatty acid, reaching a plateau at 36~~3~~ % (Supplementary Figure S2).

### *Metabolite profiling in pennycress embryos*

Along the developmental process, embryos produce a wide variety of metabolites in a temporal fashion as a result of changes in their metabolism. Metabolite profiling, also known as untargeted metabolomics, enables the detection of intracellular compounds at a given time. Through this approach one can gain qualitative (rather than quantitative) information about specific classes of intermediates accumulating at the same time as~~than~~ the synthesis of a product of interest. In this study, metabolite profiling was used to characterize all the

compounds that were present during fatty acid synthesis. For this purpose, intracellular metabolites were extracted from 17 DAP pennycress embryos with cold methanol:chloroform:water (MCW 2.5:1:1, v:v:v) (Fiehn, 2006) or boiling water (Alonso *et al.*, 2010b) and then were chemically modified with N-methyl-N-trimethylsilytrifluoroacetamide plus 1% trimethylchlorosilane (MSTFA + 1% TMCS) (Koek *et al.*, 2006). Through the comparison between GC-MS profiles of the derivatized metabolites, boiling water was shown to be the most suitable method, enabling the detection of 385 peaks versus 344 for MCW (data not shown). One hundred twelve peaks out of 385 were assigned with a probability superior or equal to 50% using the NIST 11 library (Fig. 2; Supplementary Table S1). The identification of the detected peaks qualitatively showed ~~that~~ the presence of three main classes of metabolites (sugars, amino acids, organic acids), and to a lesser extent, alkaloids, polyamines, phosphorylated metabolites and free fatty acids (Fig. 2; Supplementary Table S1).

*Comparative metabolomics analyses of developing pennycress embryos*

For the purpose of quantifying the compounds characterized by metabolite profiling, boiling water extraction was performed on pennycress embryos harvested at different stages of development. Extracted compounds were then analyzed by LC-MS/MS and quantified according to $^{13}$C-labeled internal standards as well as standard curves generated for each metabolite. The percent recovery of this method was previously determined in plant tissues for each metabolite (Cocuron *et al.*, 2014). Intermediates from ~~the~~ glycolysis, the oxidative pentose phosphate pathway (OPPP), the tricarboxylic acid cycle (TCA cycle), and Calvin cycle were measured by LC-MS/MS, indicating that all these pathways are active in developing pennycress embryos (Fig. 3; Supplementary Table S2).

Sugars are the principal source of carbon provided by the mother plant to the embryos (Allen *et al.*, 2009; Alonso *et al.*, 2010a; Alonso *et al.*, 2007; Goffman *et al.*, 2005; Lonien and Schwender, 2009; Schwender and Ohlrogge, 2002; Sriram *et al.*, 2004). Sucrose and glucose were quantified as the main free sugars in developing pennycress embryos. Their levels increased by 33-fold, from 1033.2 ± 54.3 to 33479.1 ± 1031.6 pmol embryo$^{-1}$ for glucose, and from 1161.7 ± 130.0 to 34963.7 ± 112.4 pmol embryo$^{-1}$ for sucrose (Fig. 3; Supplementary

Table S2). The main sugar alcohols were found to be sorbitol and inositol with ~~a~~ respective levels~~content~~ of 949.1 ± 281.5 and 844.8 ± 155.5 pmol embryo$^{-1}$ at 21 DAP (Fig. 3; Supplementary Table S2).

Plant embryos not only receive free amino acids as the source of nitrogen but also produce their own for protein biosynthesis (Allen *et al.*, 2009; Alonso *et al.*, 2010a; Alonso *et al.*, 2007; Goffman *et al.*, 2005; Lonien and Schwender, 2009; Schwender and Ohlrogge, 2002). The total amino acid content had a 10-fold increase in pennycress embryos between 11 and 21 DAP. Alanine, asparagine, aspartate, glutamate, glutamine, proline, and serine were the seven most abundant amino acids across ~~the~~ different developmental stages. Indeed, they represented between 83 and 90% of the total amino acids (Fig. 3; Supplementary Table S2). Besides serine that is synthesized from 3-phosphoglycerate, the six others are all produced from organic acids, at the level of the tricarboxylic acid cycle (TCA cycle; Fig. 3). The TCA cycle is also important for generating reducing power ($FADH_2$ and NADH) that can be used for biomass synthesis and/or for ATP production by oxidative phosphorylation. Malate and citrate, which are respectively involved in fatty acid synthesis and elongation (Alonso *et al.*, 2010a; Baud and Lepiniec, 2010; Fatland *et al.*, 2000; Nikolau *et al.*, 2000), reached 94% of the total organic acids at 13 DAP (Fig. 3; Supplementary Table S2).

Phosphorylated metabolites are key intermediaries of the glycolysis, OPPP, and Calvin cycle. Therefore, measuring these compounds is essential to assess central metabolism. The major phosphorylated compounds were found to be glucose 6-phosphate, fructose 6-phosphate, and pentose 5-phosphates at 11 DAP with ~~a~~ respective levels of 536.4 ± 30.5, 231.6 ± 44.7, and 206.1 ± 22.6 pmol embryo$^{-1}$ and remained high during the development of the embryo (Fig. 3; Supplementary Table S2). At 21 DAP, UDP-glucose became the most abundant phosphorylated metabolite (1112.5 ± 66.5 pmol embryo$^{-1}$) along with glucose 1-phosphate/mannose 1-phosphate (569.3 ± 30.1 pmol embryo$^{-1}$); those are major precursors for cell wall biosynthesis. Glycerol phosphate levels increased by seven-fold between 11 and 21 DAP; this metabolite provides the glycerol part of the triacylglycerols.

In order to group ~~the~~ metabolites that share a similar pattern of accumulation over ~~the~~ different stages of development, a partitional clustering analysis was

performed using MetaboAnalyst (Xia *et al.*, 2012; Xia *et al.*, 2009). Intracellular metabolites in developing pennycress embryos were found to gather in eight clusters (Fig. 4; Table 1). The majority of compounds in cluster one increased from 11 to 15 DAP and then decreased; 33% of the phosphorylated metabolites grouped into this cluster. The intermediates from cluster two, among which were nine amino acids, quickly increased to reach a plateau at 15 DAP. Clusters three, five, and seven peaked at 13, 15, and 17 DAP, respectively, and then decreased. Interestingly, fructose 1,6- bisphosphate and ribulose 1,5-bisphosphate grouped in cluster seven, and four of the organic acids in cluster five. Metabolites gradually accumulating during embryos development were gathered in cluster four; the main sugars (glucose and sucrose), several of the most abundant amino acids (glutamine and proline), and phosphorylated compounds (UDP-glucose) were found in this cluster. Fructose was the only metabolite steadily decreasing over time, and therefore apart from all the other intermediates (cluster six). Finally, all the nucleotide triphosphates grouped together in cluster eight; these had a peak at 15 DAP and then reached an optimum at 21 DAP.

## DISCUSSION:

Pennycress naturally accumulates high levels of erucic acid in its embryos, which makes it a promising biodiesel and industrial crop (Moser *et al.*, 2009a; Moser *et al.*, 2009b). Understanding the biochemical basis of oil synthesis in pennycress embryos is therefore relevant to guide future breeding and/or metabolic engineering efforts. In plants, fatty acid synthesis occurs predominantly in plastids and requires carbon (acetyl-CoA), energy (ATP) and reducing power (NADH and NADPH), which are provided *in situ* by the activity of central metabolism (Baud and Lepiniec, 2010; Hills, 2004). The main carbon sinks in pennycress embryos were found to be proteins, fatty acids, and cell wall, which respectively represented 38.5%, 33.2%, and 27.0% of the biomass at 21 DAP (Fig. 1). In comparison, the embryos of other Brassicaceae, such as *Arabidopsis thaliana* and *Physaria fendleri*, accumulate up to 40% and 55% (w/w) of oil (Cocuron *et al.*, 2014; Lonien and Schwender, 2009); these levels could be potentially achieved in pennycress too. Erucic acid reached its highest level of approximately 36~~3~~% of the total fatty acids in pennycress embryos at 19 DAP (Supplementary Figure S2). According to the results above, future crop

improvement might involve the increase of: i) the carbon flow towards ~~fatty acid~~oil synthesis, and ii) the elongation of oleic acid (C18:1) to erucic acid (C22:1). Indeed, recent genetic manipulations successfully enhanced the percentage of erucic acid in crambe (Li *et al.*, 2012), and would be a promising approach for pennycress.

Metabolomics emerged as a powerful tool to assess the metabolic state of a given organism/tissue (Patti *et al.*, 2012), specifically quantifying key intermediary compounds involved in primary pathways (Alonso *et al.*, 2010b; Bajad *et al.*, 2006; Cocuron and Alonso, 2014; Cocuron *et al.*, 2014; Huck *et al.*, 2003; Koubaa *et al.*, 2013; Luo *et al.*, 2007). In this study, intracellular metabolites were extracted from pennycress embryos using boiling water, which has been shown to be the most efficient method to extract water-soluble compounds from various biological sources (micro-organisms, mammalian cells, plant tissues, etc) with the maximum recovery (Alonso *et al.*, 2010a; Cocuron *et al.*, 2014; El Rammouz *et al.*, 2010). Two metabolomic approaches were applied in this work to fingerprint the physiological activities of pennycress embryos. The first one, untargeted metabolomics, is a ~~method~~ purely qualitative method: it has been widely used to assess the global metabolite profile of a sample and to detect novel entities (Koek *et al.*, 2006; Macel *et al.*, 2014; Mie *et al.*, 2014; Patti *et al.*, 2012; Wolfender *et al.*, 2013). In this study, metabolite fingerprinting and the main classes of intermediaries in pennycress embryos were determined using GC-MS and a structural database (Fig. 2). The second approach is more targeted and relies on the selective quantification of key intracellular metabolites. The levels of intermediaries may be expressed as relative (Borisjuk *et al.*, 2013; Rolletschek *et al.*, 2011) or absolute values (Cocuron *et al.*, 2014; Urakami *et al.*, 2010; Wu *et al.*, 2014); with absolute quantities offering the possibility to draw comparisons between different metabolites, in various tissues, conditions, etc. High-throughput LC-MS/MS methods have been recently developed and validated to separate and quantify the intermediaries and precursors for plant biomass synthesis: amino acids, sugars/sugar alcohols, phosphorylated compounds, and organic acids, which represents about 100 metabolites (Alonso *et al.*, 2010b; Cocuron and Alonso, 2014; Cocuron *et al.*, 2014; Koubaa *et al.*, 2013). These methods have been applied here to compare intracellular metabolite levels in pennycress embryos at different stages of development (Figure 3,

Supplementary Table S2). It is important to note that the targeted metabolomics study presented here was performed with the future objective to carry out flux analysis. In the plant field, it is the norm to describe units in per embryo basis for flux analysis and in the future it will be useful in comparing the net accumulation of intermediary metabolites to the carbon fluxes through the metabolic pathways. However, metabolite quantities have also been reported here in per mg DW (Supplementary Table S3) in order to facilitate the comparison with other plants, organs, etc.

In this study, metabolomics was used to probe the activity of central metabolic pathways and to determine their implication in fatty acid synthesis. First, the main intracellular sugars and amino acids were found to be sucrose, glucose, and glutamine (Fig. 3, Supplementary Table S2), which were grouped into cluster four (Fig. 4; Table 1); these are respectively common sources of carbon and nitrogen for developing plant embryos (Allen *et al.*, 2009; Alonso *et al.*, 2010a; Alonso *et al.*, 2007; Goffman *et al.*, 2005; Lonien and Schwender, 2009; Schwender and Ohlrogge, 2002; Sriram *et al.*, 2004). In developing pennycress embryos, fructose was the only metabolite in cluster six (Fig. 4; Table 1) with its level decreasing overtime. This observation, together with the high levels of sucrose and glucose, indicate that sucrose might be stored rather than cleaved into hexoses via the invertase. In agreement with this study, targeted metabolomics on the embryos of another Brassicaceae, *Physaria fendleri*, also reported the accumulation of sucrose across developmental stages (Cocuron *et al.*, 2014).-A variety of integrative functions have been suggested for sucrose storage in plant embryos including modulation of gene expression, protein turnover and a trigger to induce storage pathway (Borek and Nuc, 2011; Farrar *et al.*, 2000; Weber *et al.*, 1997). In earlier studies, storage activity was shown to occur in both avocado and field bean embryos when sucrose level increased (Sanchez-Romero *et al.*, 2002; Weber *et al.*, 1997). Additionally, sucrose storage is involved in the acquisition of desiccation tolerance during seed development and maturation (Businge *et al.*, 2013). Sugars received by the embryos are metabolized in the cytosol, supplying carbon skeletons to biomass synthesis (Baud and Lepiniec, 2010; Hills, 2004). Second, the main organic acids were malate and citrate (Fig. 3, Supplementary Table S2) which have been shown to provide acetyl-CoA for fatty acid synthesis and elongation, respectively

(Alonso *et al.*, 2010a; Baud and Lepiniec, 2010; Fatland *et al.*, 2000; Nikolau *et al.*, 2000). Third, the presence of ribulose 1,5-bisphosphate (Figure 3, Supplementary Table S2), a metabolite specific to the Calvin cycle, and the green color of the embryos (Fig. 1) indicate that they are photosynthetically active between 11 and 21 DPA. It has been observed that in green seeds, light energy can be used by chloroplasts to generate ATP and NADPH (Browse and Slack, 1985; Goffman *et al.*, 2005; Ohlrogge *et al.*, 2004; Schwender *et al.*, 2004; Schwender *et al.*, 2006). Therefore photosynthesis might provide part of the energy and reductant necessary for fatty acid production in developing pennycress embryos. Furthermore, ribulose 1,5-bisphosphate is the substrate of the ribulose 1,5-bisphosphate carboxylase/oxygenase (RuBisCo). High activities of this enzyme have been measured in Brassicaceae embryos (King *et al.*, 1998; Ruuska *et al.*, 2004) where it has been shown to fix the $CO_2$ released by the pyruvate dehydrogenase, increasing the efficiency of carbon use (Schwender *et al.*, 2004). Interestingly ribulose 1,5-bisphosphate uniquely clustered with fructose 1,6-bisphosphate (Fig. 4; Table 1), which indicates that a large portion of the fructose 1,6-bisphosphate might be produced by the Calvin cycle. Finally, the mitochondrial respiration and OPPP usually are the two other pathways generating ATP and NADPH, respectively. The level of free amino acids was found to be higher than their direct precursors from glycolysis, TCA cycle and OPPP (Fig. 3; Supplemental Table S2), revealing a high flow of carbon through these pathways. Besides photosynthesis, oxidative phosphorylation and OPPP might be a significant source of energy and reductant for oil synthesis in developing pennycress embryos.

To date, there is only another one quantitative metabolomics study which was conducted on developing embryos of *Physaria fendleri* (Cocuron *et al.*, 2014). The major developmental difference between Physaria and pennycress embryos was the rate of DW accumulation being twice faster in pennycress. The comparison of the intracellular compound levels between same stage embryos (*ie.* 17 and 27 DAP for pennycress and Physaria, respectively) highlighted major biochemical and metabolic differences. First, Physaria embryos synthesized more oil (55% *vs.* 33%; w/w). Second, besides sucrose, the levels of the other major free sugars were higher whereas hexose-phosphates were lower in Physaria, suggesting a faster glycolytic flow in pennycress embryos. Third, the

amounts of all the organic acids were lower in Physaria by a factor six to 55, which may indicate a slower TCA cycle. Finally, the intermediaries of the OPPP (6-phosphogluconate, sedoheptulose 7-phosphate, pentose-phosphates) and Calvin cycle (ribulose 1,5-bisphosphate) were found to be higher in pennycress embryos, suggesting a larger flow of carbon through these pathways. Metabolomics study hence revealed the occurrence of key pathways involved in oil production in pennycress embryos. However, the relative contribution of each of these pathways to the synthesis of fatty acids (in terms of carbon skeletons, energy, and reductant), and the potential bottlenecks can only be determined by measuring the *in vivo* metabolic fluxes (Alonso *et al.*, 2010a; Dieuaide-Noubhani and Alonso, 2014).

**SUPPLEMENTARY DATA:**

*Supplementary Fig. S1: Thlaspi arvense L. plant anatomy.*

*Supplementary Fig. S2: Fatty acid composition in developing pennycress embryos.*

*Supplementary Table S1: Untargeted metabolomics analysis of pennycress embryos at 17 DAP.*

*Supplementary Table S2: Targeted metabolomics analyses of pennycress embryos at different developmental stages.*

*Supplementary Table S3: Metabolite levels in developing pennycress embryos expressed in pmol mg DW$^{-1}$*

**ACKNOWLEDGEMENTS:**

M.C. was supported by a REU summer training grant to OSU (DBI-1062144). We are grateful to The Ohio State University Targeted Metabolomics Laboratory (metabolomics.osu.edu) for access to the GC-MS and LC-MS/MS equipments funded respectively by the Center for Applied Plant Sciences (CAPS) and the Translational Plant Sciences Targeted Investment in Excellence (TIE). We thank

Brooke Anderson for technical help as well as Gary Posey (Greenhouse superintendent).

**REFERENCES:**

**Allen DK, Ohlrogge JB, Shachar-Hill Y**. 2009. The role of light in soybean seed filling metabolism. *Plant Journal* **58**, 220-234.

**Alonso AP, Dale VL, Shachar-Hill Y**. 2010a. Understanding fatty acid synthesis in developing maize embryos using metabolic flux analysis. *Metabolic Engineering* **12**, 488-497.

**Alonso AP, Goffman FD, Ohlrogge JB, Shachar-Hill Y**. 2007. Carbon conversion efficiency and central metabolic fluxes in developing sunflower (Helianthus annuus L.) embryos. *Plant Journal* **52**, 296-308.

**Alonso AP, Piasecki RJ, Wang Y, LaClair RW, Shachar-Hill Y**. 2010b. Quantifying the Labeling and the Levels of Plant Cell Wall Precursors Using Ion Chromatography Tandem Mass Spectrometry. *Plant physiology* **153**, 915-924.

**Bajad SU, Lu W, Kimball EH, Yuan J, Peterson C, Rabinowitz JD**. 2006. Separation and quantitation of water soluble cellular metabolites by hydrophilic interaction chromatography-tandem mass spectrometry. *Journal of chromatography. A* **1125**, 76-88.

**Baud S, Lepiniec L**. 2010. Physiological and developmental regulation of seed oil production. *Progress in Lipid Research* **49**, 235-249.

**Baud S, Mendoza MS, To A, Harscoet E, Lepiniec L, Dubreucq B**. 2007. WRINKLED1 specifies the regulatory action of LEAFY COTYLEDON2 towards fatty acid metabolism during seed maturation in Arabidopsis. *The Plant journal : for cell and molecular biology* **50**, 825-838.

**Boateng AA, Mullen CA, Goldberg NM**. 2010. Producing Stable Pyrolysis Liquids from the Oil-Seed Presscakes of Mustard Family Plants: Pennycress (Thlaspi arvense L.) and Camelina (Camelina sativa). *Energy & Fuels* **24**, 6624-6632.

**Borek S, Nuc K**. 2011. Sucrose controls storage lipid breakdown on gene expression level in germinating yellow lupine (Lupinus luteus L.) seeds. *Journal of Plant Physiology* **168**, 1795-1803.

**Borisjuk L, Neuberger T, Schwender J, Heinzel N, Sunderhaus S, Fuchs J, Hay JO, Tschiersch H, Braun HP, Denolf P, Lambert B, Jakob PM, Rolletschek H**. 2013. Seed architecture shapes embryo metabolism in oilseed rape. *The Plant cell* **25**, 1625-1640.

**Bouaid A, Martinez M, Aracil J**. 2009. Production of biodiesel from bioethanol and Brassica carinata oil: Oxidation stability study. *Bioresource Technology* **100**, 2234-2239.

**Browse J, Slack CR**. 1985. Fatty-Acid Synthesis in Plastids from Maturing Safflower and Linseed Cotyledons. *Planta* **166**, 74-80.

**Businge E, Bygdell J, Wingsle G, Moritz T, Egertsdotter U**. 2013. The effect of carbohydrates and osmoticum on storage reserve accumulation and germination of Norway spruce somatic embryos. *Physiologia Plantarum* **149**, 273-285.

**Cermak SC, Biresaw G, Isbell TA, Evangelista RL, Vaughn SF, Murray R**. 2013. New crop oils-Properties as potential lubricants. *Industrial Crops and Products* **44**, 232-239.

**Cocuron JC, Alonso AP**. 2014. Liquid chromatography tandem mass spectrometry for measuring 13C-labeling in intermediaries of the glycolysis and pentose-phosphate pathway. *Methods in Molecular Biology* **1090**, 131-142.

**Cocuron JC, Anderson B, Boyd A, Alonso AP**. 2014. Targeted metabolomics of *Physeria fendleri*, an industrial crop producing hydroxy fatty acids. *Plant and Cell Physiology* **55**, 620-633.

**Demirbas A**. 2009. Political, economic and environmental impacts of biofuels: A review. *Applied Energy* **86**, S108-S117.

**Dieuaide-Noubhani M, Alonso AP**. 2014. Application of metabolic flux analysis to plants. *Methods in Molecular Biology* **1090**, 1-18.

**El Rammouz R, Letisse F, Durand S, Portais JC, Moussa ZW, Fernandez X**. 2010. Analysis of skeletal muscle metabolome: evaluation of extraction methods for targeted metabolite quantification using liquid chromatography tandem mass spectrometry. *Analytical Biochemistry* **398**, 169-177.

**Fan JQ, Shonnard DR, Kalnes TN, Johnsen PB, Rao S**. 2013. A life cycle assessment of pennycress (Thlaspi aruense L.) -derived jet fuel and diesel. *Biomass & Bioenergy* **55**, 87-100.

**Farrar J, Pollock C, Gallagher J**. 2000. Sucrose and the integration of metabolism in vascular plants. *Plant Science* **154**, 1-11.

**Fatland B, Anderson M, Nikolau BJ, Wurtele ES**. 2000. Molecular biology of cytosolic acetyl-CoA generation. *Biochemical Society Transactions* **28**, 593-595.

**Fiehn O**. 2006. Metabolite profiling in Arabidopsis. *Methods in Molecular Biology* **323**, 439-447.

**Fiehn O**. 2008. Extending the breadth of metabolite profiling by gas chromatography coupled to mass spectrometry. *Trends in analytical chemistry : TRAC* **27**, 261-269.

**Frohlich A, Rice B**. 2005. Evaluation of Camelina sativa oil as a feedstock for biodiesel production. *Industrial Crops and Products* **21**, 25-31.

**Goffman FD, Alonso AP, Schwender J, Shachar-Hill Y, Ohlrogge JB**. 2005. Light enables a very high efficiency of carbon storage in developing embryos of rapeseed. *Plant physiology* **138**, 2269-2279.

**Hills MJ**. 2004. Control of storage-product synthesis in seeds. *Current opinion in plant biology* **7**, 302-308.

**Hojilla-Evangelista MP, Evangelista RL, Isbell TA, Selling GW**. 2013. Effects of cold-pressing and seed cooking on functional properties of protein in pennycress (Thlaspi arvense L.) seed and press cakes. *Industrial Crops and Products* **45**, 223-229.

**Huck JHJ, Struys EA, Verhoeven NM, Jakobs C, Van der Knaap MS**. 2003. Profiling of pentose phosphate pathway intermediates in blood spots by tandem mass spectrometry: Application to transaldolase deficiency. *Clinical Chemistry* **49**, 1375-1380.

**Isbell TA**. 2009. US effort in the development of new crops (Lesquerella, Pennycress, Coriander and Cuphea). *Oleagineux Corps Gras Lipides* **16**, 205-210.

**Kim S, Dale BE**. 2005. Life cycle assessment of various cropping systems utilized for producing biofuels: Bioethanol and biodiesel. *Biomass & Bioenergy* **29**, 426-439.

**King WA, Gready JE, Andrews TJ**. 1998. Quantum chemical analysis of the enolization of ribulose bisphosphate: the first hurdle in the fixation of CO2 by Rubisco. *Biochemistry* **37**, 15414-15422.

**Koek MM, Muilwijk B, van der Werf MJ, Hankemeier T**. 2006. Microbial metabolomics with gas chromatography/mass spectrometry. *Analytical chemistry* **78**, 1272-1281.

**Koubaa M, Cocuron J-C, Thomasset B, Alonso AP**. 2013. Highlighting the tricarboxilic acid cycle: liquid and gas chromatography-mass spectrometry analyses of 13C-labeled organic acids. *Analytical Biochemistry* **436**, 151-159.

**Li XY, van Loo EN, Gruber J, Fan J, Guan R, Frentzen M, Stymne S, Zhu LH**. 2012. Development of ultra-high erucic acid oil in the industrial oil crop Crambe abyssinica. *Plant biotechnology journal* **10**, 862-870.

**Lonien J, Schwender J**. 2009. Analysis of metabolic flux phenotypes for two Arabidopsis mutants with severe impairment in seed storage lipid synthesis. *Plant physiology* **151**, 1617-1634.

**Luo B, Groenke K, Takors R, Wandrey C, Oldiges M**. 2007. Simultaneous determination of multiple intracellular metabolites in glycolysis, pentose phosphate pathway and tricarboxylic acid cycle by liquid chromatography-mass spectrometry. *Journal of Chromatography A* **1147**, 153-164.

**Macel M, de Vos RC, Jansen JJ, van der Putten WH, van Dam NM**. 2014. Novel chemistry of invasive plants: exotic species have more unique metabolomic profiles than native congeners. *Ecology and evolution* **4**, 2777-2786.

**Mie A, Laursen KH, Aberg KM, Forshed J, Lindahl A, Thorup-Kristensen K, Olsson M, Knuthsen P, Larsen EH, Husted S**. 2014. Discrimination of conventional and organic white cabbage from a long-term field trial study using untargeted LC-MS-based metabolomics. *Analytical and bioanalytical chemistry* **406**, 2885-2897.

**Moser BR, Knothe G, Vaughn SF, Isbell TA**. 2009a. Production and Evaluation of Biodiesel from Field Pennycress (Thlaspi arvense L.) Oil. *Energy & Fuels* **23**, 4149-4155.

**Moser BR, Shah SN, Winkler-Moser JK, Vaughn SF, Evangelista RL**. 2009b. Composition and physical properties of cress (Lepidium sativum L.) and field pennycress (Thlaspi arvense L.) oils. *Industrial Crops and Products* **30**, 199-205.

**Nikolau BJ, Oliver DJ, Schnable PS, Wurtele ES**. 2000. Molecular biology of acetyl-CoA metabolism. *Biochemical Society Transactions* **28**, 591-593.

**Ogura T, Bamba T, Fukusaki E**. 2013. Development of a practical metabolite identification technique for non-targeted metabolomics. *Journal of Chromatography A* **1301**, 73-79.

**Ohlrogge JB, Ruuska SA, Schwender J**. 2004. The capacity of green oilseeds to utilize photosynthesis to drive biosynthetic processes. *Plant physiology* **136**, 2700-2709.

**Patti GJ, Yanes O, Siuzdak G**. 2012. Innovation: Metabolomics: the apogee of the omics trilogy. *Nature reviews. Molecular cell biology* **13**, 263-269.

**Phippen WB, Phippen ME**. 2012. Soybean Seed Yield and Quality as a Response to Field Pennycress Residue. *Crop Science* **52**, 2767-2773.

**Robson P, Jensen E, Hawkins S, White SR, Kenobi K, Clifton-Brown J, Donnison I, Farrar K**. 2013. Accelerating the domestication of a bioenergy crop: identifying and modelling morphological targets for sustainable yield increase in Miscanthus. *Journal of Experimental Botany* **64**, 4143-4155.

**Rolletschek H, Melkus G, Grafahrend-Belau E, Fuchs J, Heinzel N, Schreiber F, Jakob PM, Borisjuk L**. 2011. Combined noninvasive imaging and modeling approaches reveal metabolic compartmentation in the barley endosperm. *The Plant cell* **23**, 3041-3054.

**Ruuska SA, Schwender J, Ohlrogge JB**. 2004. The capacity of green oilseeds to utilize photosynthesis to drive biosynthetic processes. *Plant physiology* **136**, 2700-2709.

**Sanchez-Romero C, Peran-Quesada R, Barcelo-Munoz A, Pliego-Alfaro F**. 2002. Variations in storage protein and carbohydrate levels during development of avocado zygotic embryos. *Plant Physiology and Biochemistry* **40**, 1043-1049.

**Schwender J, Goffman F, Ohlrogge JB, Shachar-Hill Y**. 2004. Rubisco without the Calvin cycle improves the carbon efficiency of developing green seeds. *Nature* **432**, 779-782.

**Schwender J, Ohlrogge JB**. 2002. Probing in vivo metabolism by stable isotope labeling of storage lipids and proteins in developing Brassica napus embryos. *Plant physiology* **130**, 347-361.

**Schwender J, Shachar-Hill Y, Ohlrogge JB**. 2006. Mitochondrial metabolism in developing embryos of Brassica napus. *Journal of Biological Chemistry* **281**, 34040-34047.

**Sriram G, Fulton DB, Iyer VV, Peterson JM, Zhou R, Westgate ME, Spalding MH, Shanks JV**. 2004. Quantification of compartmented metabolic fluxes in developing soybean embryos by employing biosynthetically directed fractional (13)C labeling, two-dimensional [(13)C, (1)H] nuclear magnetic

resonance, and comprehensive isotopomer balancing. *Plant physiology* **136**, 3043-3057.

**Urakami K, Zangiacomi V, Yamaguchi K, Kusuhara M**. 2010. Quantitative metabolome profiling of Illicium anisatum by capillary electrophoresis time-of-flight mass spectrometry. *Biomedical research* **31**, 161-163.

**Vaughn SF, Isbell TA, Weisleder D, Berhow MA**. 2005. Biofumigant compounds released by field pennycress (Thlaspi arvense) seedmeal. *Journal of chemical ecology* **31**, 167-177.

**Warwick SI, Francis A, Susko DJ**. 2002. The biology of Canadian weeds. 9. Thlaspi arvense L. (updated). *Canadian Journal of Plant Science* **82**, 803-823.

**Weber H, Borisjuk L, Wobus U**. 1997. Sugar import and metabolism during seed development. *Trends in plant science* **2**, 169-174.

**Wolfender JL, Rudaz S, Choi YH, Kim HK**. 2013. Plant metabolomics: from holistic data to relevant biomarkers. *Current medicinal chemistry* **20**, 1056-1090.

**Wu X, Li N, Li H, Tang H**. 2014. An optimized method for NMR-based plant seed metabolomic analysis with maximized polar metabolite extraction efficiency, signal-to-noise ratio, and chemical shift consistency. *The Analyst* **139**, 1769-1778.

**Xia J, Mandal R, Sinelnikov IV, Broadhurst D, Wishart DS**. 2012. MetaboAnalyst 2.0--a comprehensive server for metabolomic data analysis. *Nucleic acids research* **40**, W127-133.

**Xia J, Psychogios N, Young N, Wishart DS**. 2009. MetaboAnalyst: a web server for metabolomic data analysis and interpretation. *Nucleic acids research* **37**, W652-660.

**TABLES:**

*Table 1: Clusters of metabolites in developing pennycress embryos.*

Metabolites were clustered using MetaboAnalyst v2.5.

| Metabolites | | | | |
|---|---|---|---|---|
| **Cluster** | **Sugars & sugar alcohols** | **Amino acids** | **Phosphorylated compounds** | **Organic acids** |
| **1** | INO | Arg, Ala, His, GABA, OHPro | PGA, F6P, G6P, ADP, IMP, CDP, Gal1P, GLYP, isoCIT, UDP, P5P, | isoCIT~~Arg, Ala, His, GABA, OHPro~~ |
| **2** | | Glu, Gly, Lys, Met, Ser, Thr, Tyr, Val | CIT, GDP, GMP, Man6P, MAL, UMP, SUCP | MAL~~Glu, Gly, Lys, Met, Ser, Thr, Tyr, Val~~ |
| **3** | | Asn | 6PG, dX5P, S7P, PEP | ~~Asn~~ |
| **4** | Glc, Sorb, Ery/Thr, Pentitols, SUC | Cys, Phe, Pro, Gln, Ile | ~~transACO,~~ UDPG, CMP, AMP, ~~FUM, SHI~~ | transACO, FUM, SHI ~~Cys, Phe, Pro, Gln, Ile~~ |
| **5** | | | F1,6bP, R1,5bP | |
| **6** | FRU | | | |
| **7** | | | ~~AKG, cisACO, CIT, SUCC~~, T6P, G1P | AKG, cisACO, CIT, SUCC |

| | | | |
|---|---|---|---|
| **8** | Asp, ~~Orn~~ | ATP, CTP, GTP, UTP | ~~Asp,~~ Orn |

## FIGURE LEGENDS:

*Figure 1: Biomass composition of pennycress embryos at different stages of development.*

A) Pictures of the embryos at different stages of development at the time of dissection under a binocular microscope. B) Biomass accumulation rate of pennycress embryos. The orange circles, purple squares, green squares, red triangles, and blue triangles respectively represent the dry weight~~ whereas the blue diamonds, green squares and red triangles depict, respectively~~, the amounts of protein~~s~~, fatty acid~~s, and ~~starch, and cell wall accumulating into a pennycress embryo (n=4 biological replicates). C) Biomass abundance in pennycress embryo. The purple, green,~~ and~~ red and blue bars are associated, respectively, with the percentage (w/w) of protein, fatty acid,~~ and~~ starch, and cell wall characterizing a single embryo. Error bars are the SD of four biological replicates.

*Figure 2: Metabolite profiling of pennycress embryos at 17 DAP.*

A) GC-MS chromatogram of 17 DAP pennycress embryos obtained after MSTFA derivatization. Enlarged chromatogram areas depicting the main classes of compounds, B) amino acids, C) organic acids and D) sugars found in pennycress embryos. NIST 11 library was used to assign the different peaks.

*Figure 3: Metabolic map of pennycress embryos at different stages of development.*

Values are expressed in pmol per embryo (pmol/emb) and are the average ± SD of three biological replicates from embryos harvested at 11, 13, 15, 17, 19 and 21 DAP. SUC, sucrose; FRU, fructose; GLC, glucose; INO, inositol; GLY, glycerol; Ery/Thr, erythritol/threitol; Ala, alanine; Arg, arginine; Asn, asparagine; Asp, aspartate; Cys, cysteine; Lys, lysine; Gln, glutamine; Glu, glutamate; Gly, glycine; His, histidine; OHPro, hydroxyproline; Leu, leucine; Ile, isoleucine; Met, methionine; Phe, phenylalanine; Pro, proline; Ser, serine; Thr, threonine; Tyr, tyrosine; Val, valine; GABA, 4-aminobutyric acid; Orn, ornithine; Citru, citrulline; T6P, trehalose 6-phosphate; UDPG, UDP-glucose; SUCP, sucrose 6-phosphate; G1P, glucose 1-phosphate; M1P/G1P, mannose 1-phosphate/glucose 1-phosphate; F6P, fructose 6-phosphate; G6P, glucose 6-

phosphate; 6PG, 6-phosphogluconic acid; P5P, pentose 5-phosphate; R1,5-bP, ribulose 1,5-bisphosphate; S7P, sedoheptulose 7-phosphate; E4P, eryhtrose 4-phosphate; F1,6bP, fructose 1,6-bisphosphate; GLYP, glycerol-phosphates; TP, triose phosphates; PGA, 2-3 phosphoglycerates; dX5P, deoxyxylulose 5-phosphate; PEP, phosphoenolpyruvate; SHI, shikimate; PYR, pyruvate; AcCoA, acetyl-CoA; CIT, citrate; cisACO, cis-aconitate; isoCIT, isocitrate; AKG, α-ketoglutarate; SUCC, succinate; FUM, fumarate; MAL, malate; OAA, oxaloacetate. Metabolites colored in red and green correspond to glycolysis and TCA cycle, respectively.

*Figure 4: Metabolite clustering of pennycress embryos across different developmental stages.*

Metabolites were clustered using MetaboAnalyst v2.5. The black lines represent median intensities of corresponding clusters that were obtained from K-means analysis.

**FIGURES:**

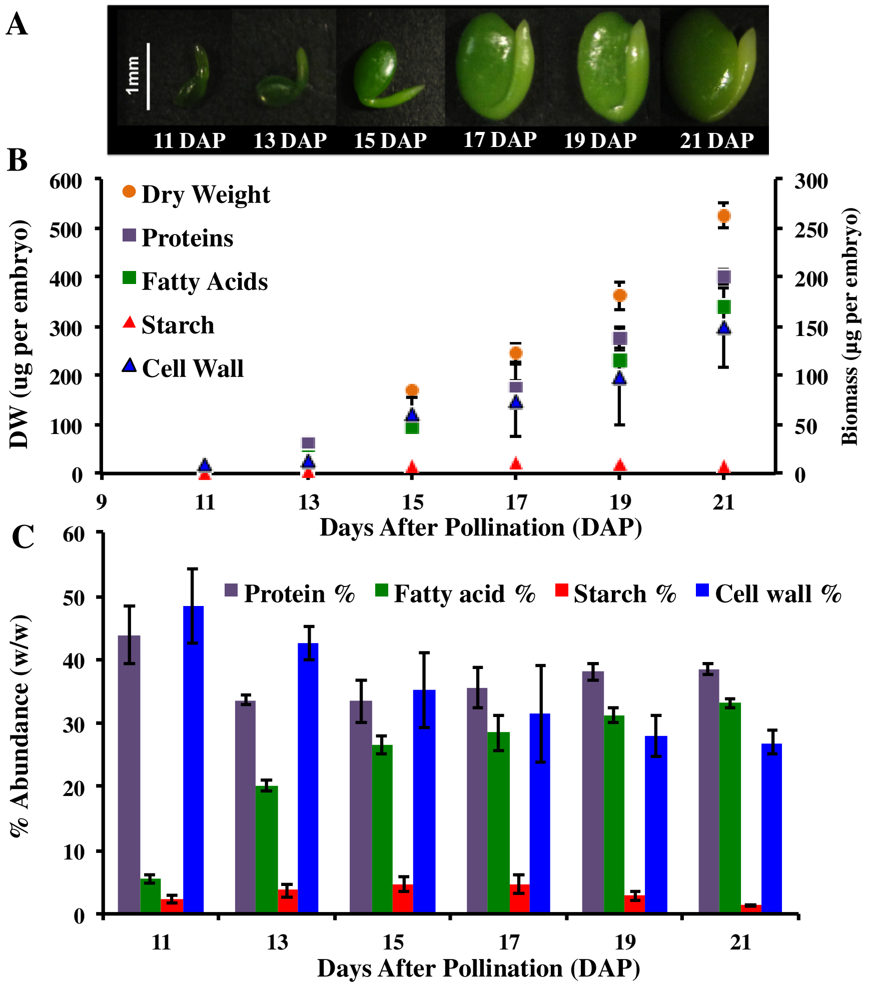


*Figure 1: Biomass composition of pennycress embryos at different stages of development.*

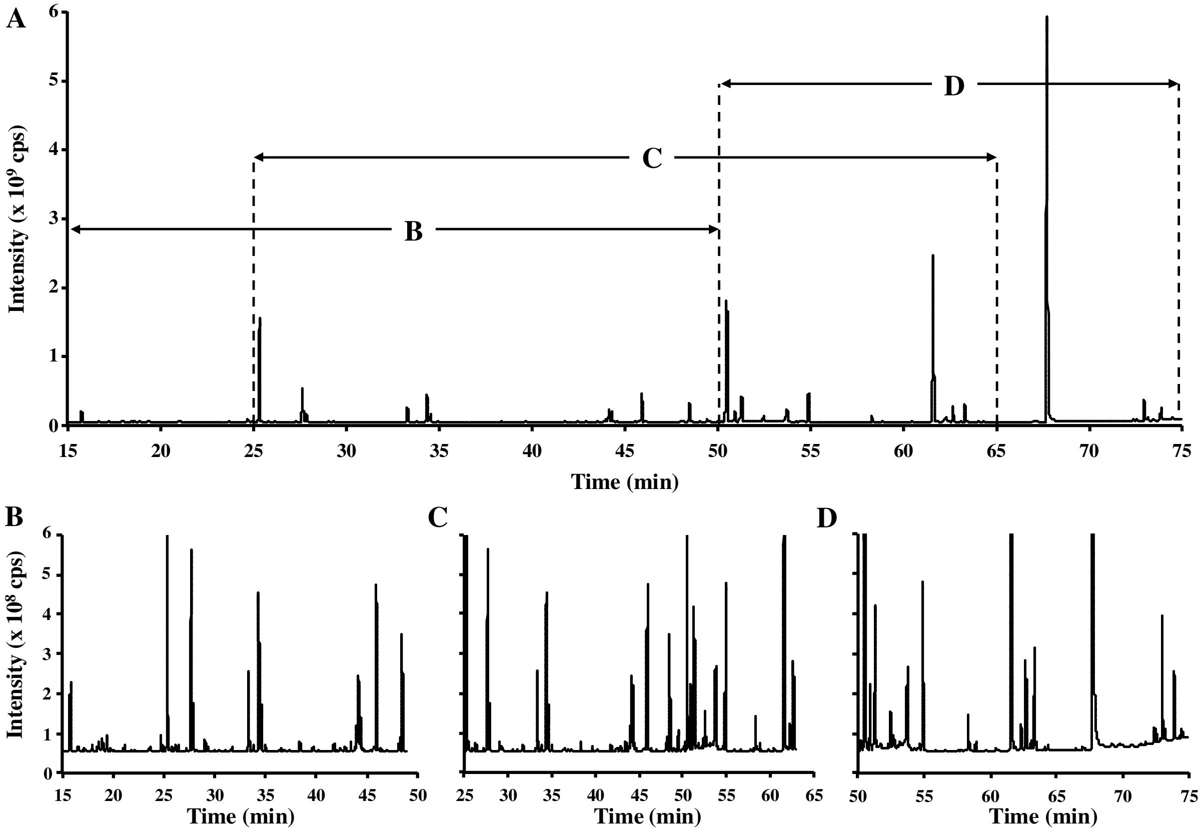


*Figure 2: Metabolite profiling of pennycress embryos at 17 DAP.*

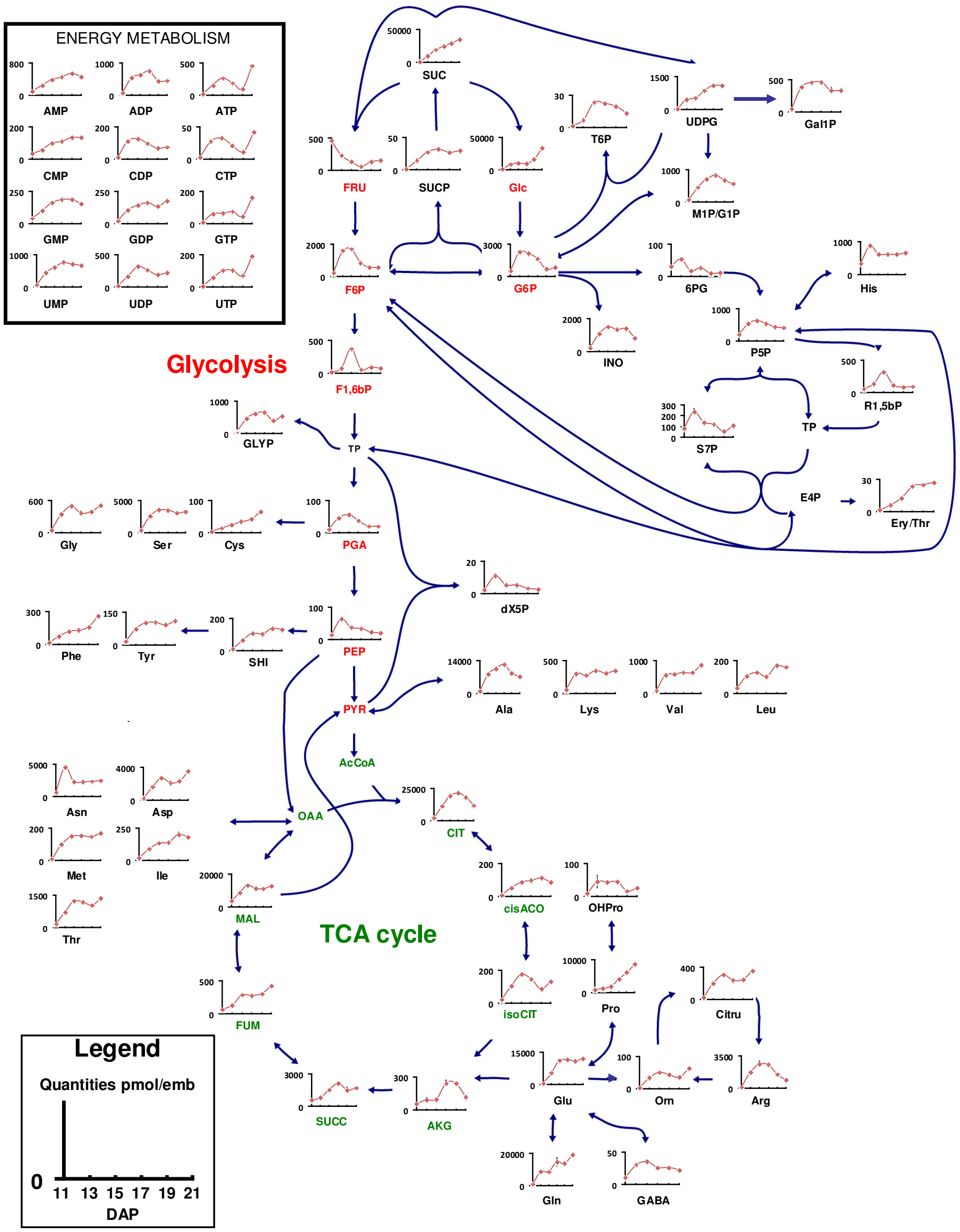


*Figure 3: Metabolic map of pennycress embryos at different stages of development.*

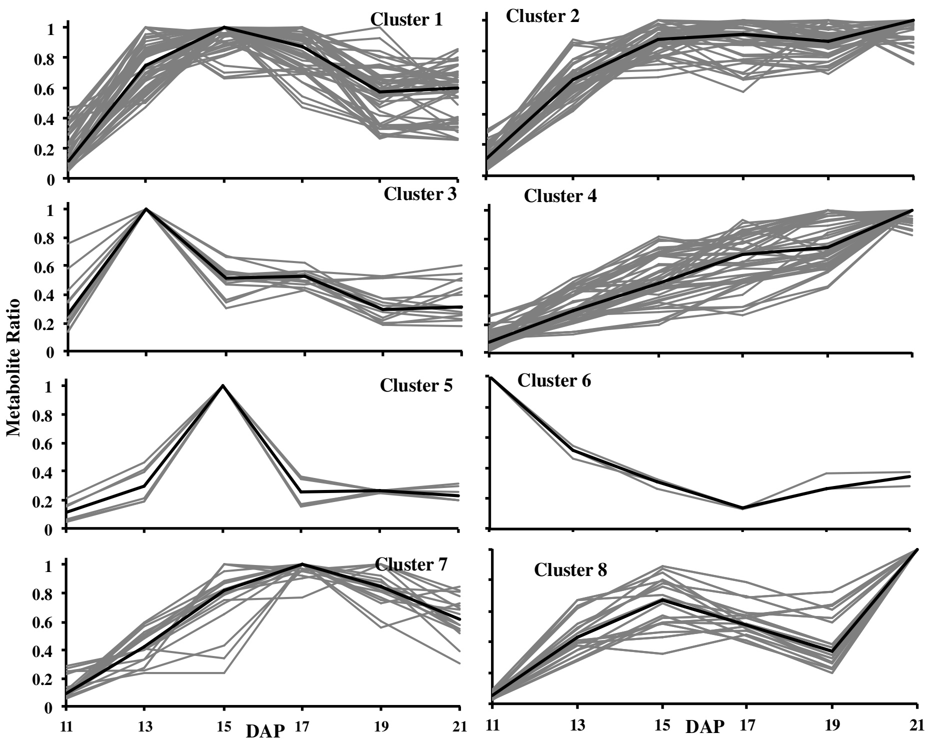


*Figure 4: Metabolite clustering of pennycress embryos across different developmental stages.*

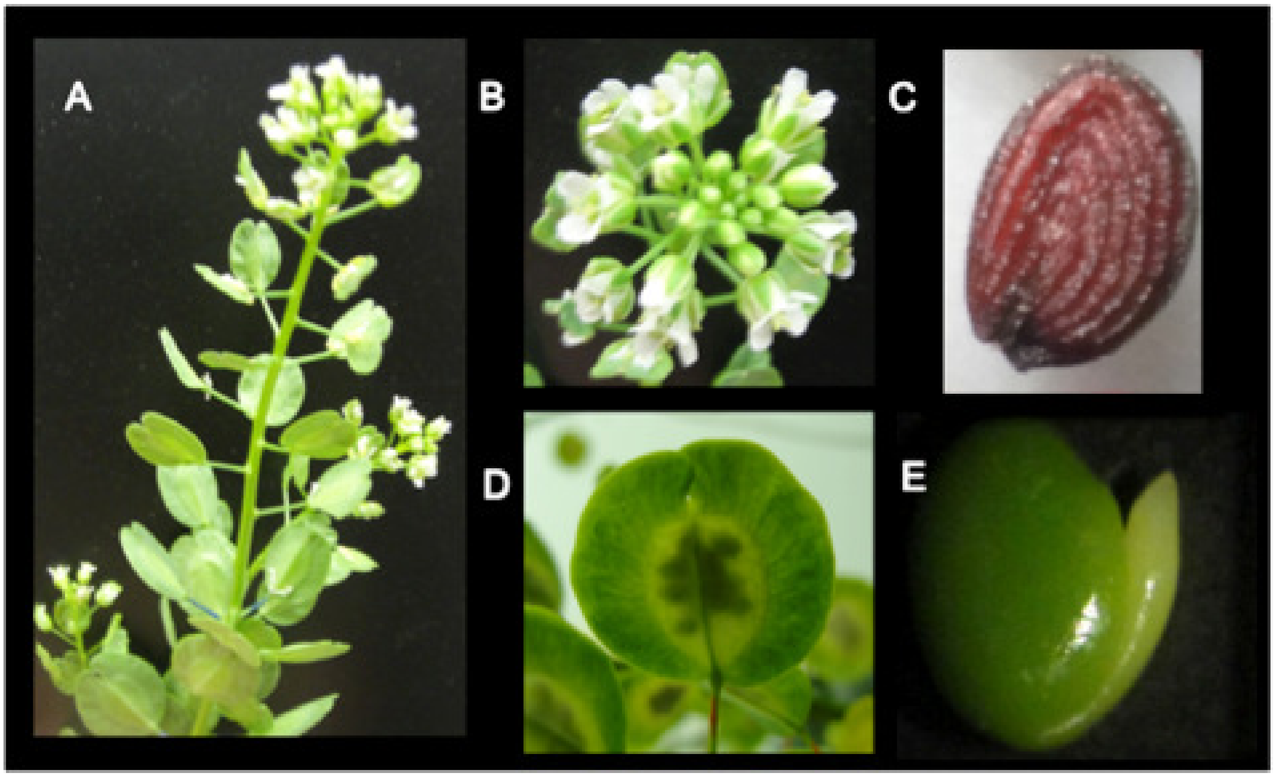


**Supplementary Figure S1.** *Thlaspi arvense L.* plant anatomy. **A**) Mature Plant; **B**) Flower; **C**) Mature seed; **D**) Silique; **E**) Embryo.

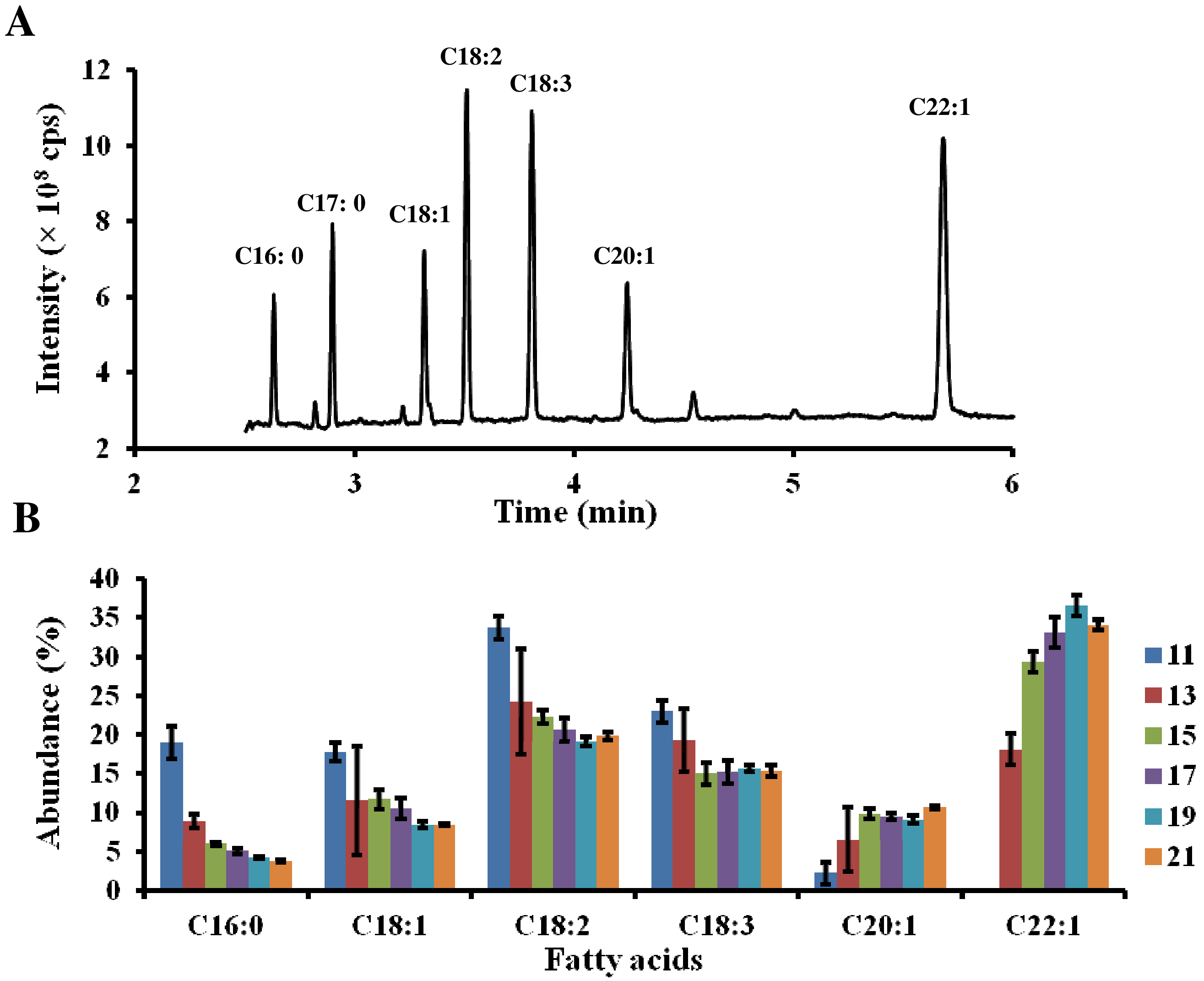


**Supplementary Figure S2**. **Fatty acid composition in developing pennycress embryos.** **A**) GC-MS profile of total fatty acid composition in embryo at 17 DAP. C16:0, palmitic; C17:0, margaric as an internal standard; C18:1, oleic; C18:2 linoleic; C18:3, linolenic; C20:1, eicosenoic; C22:1, erucic acid. **B**) The percentage abundance of the major fatty acids in embryos. Error bars represent the SD of four biological replicates. The blue, red, green, purple, turquoise and orange bars are the percentage of each fatty acid at 11, 13, 15, 17, 19 and 21 DAP stages, respectively.

**Supplemental table 1. Untargeted metabolomics analysis of pennycress embryo at different developmental stages using GC-MS.**
Metabolites listed below were identified using NIST 11 library with at least a 50 % probability. Profiling of pennycress embryos was carried at 11, 17 and 21 DPA stages (n=2 biological replicates).

| Metabolite name | Retention time (min) |
|---|---|
| Glycolic acid | 14.5 |
| Alanine | 15.8 |
| Glycolic acid | 16.5 |
| Dihydroquinoline | 19.0 |
| Hyroxysimazine | 19.2 |
| Ethoxyquin | 21.0 |
| Valine | 21.2 |
| Benzoic acid | 22.2 |
| Nicotinic acid | 24.3 |
| Proline | 24.9 |
| Glycine | 25.3 |
| Succinic acid | 25.5 |
| 6-deoxyhexose | 25.9 |
| Glyceric acid | 26.6 |
| Isocyanic acid derivative | 27.0 |
| Caprylic acid | 27.3 |
| 4-Nitrobenzoic acid | 27.7 |
| Serine | 27.9 |
| Threonine | 29.1 |
| beta-Alanine | 30.5 |
| Homoserine | 31.7 |
| Coumaric acid | 32.5 |
| Malic acid | 33.3 |
| Adipic acid | 33.6 |
| Methyl Octanoic acid | 33.8 |
| Pyroglutamic acid | 34.4 |
| Aspartic aicd | 34.6 |
| Threonic acid | 36.5 |
| Pentose | 37.3 |
| Pentose | 38.2 |
| Glutamic acid | 38.4 |
| D-Ribonic acid- γ-lactone | 39.7 |
| L-Asparagine | 40.3 |
| Pentose | 41.8 |
| N-Acetyl-lysine | 42.4 |
| Hexose | 43.5 |
| Putrescine | 42.6 |
| Hexose | 42.9 |
| Disaccharide | 43.6 |
| Pentitol | 43.9 |
| Ribulose-5-phosphate | 44.0 |
| Pentose | 44.2 |
| Hexose | 44.4 |
| Ornithine | 45.6 |
| Isocitric/Citric acid | 45.9 |
| Pentose | 46.2 |
| Pentose | 46.4 |
| Hexose | 47.0 |
| Asparagine | 47.4 |
| Hexose | 48.1 |
| Hexose | 48.3 |
| Hexose | 48.5 |
| Hexose | 48.6 |
| Lysine | 49.1 |
| Hexose | 49.5 |
| Hexose | 50.1 |

| Metabolite name | Retention time (min) |
|---|---|
| Tetraose | 50.2 |
| Absorbic acid | 50.3 |
| Hexose | 50.5 |
| Hexose | 50.9 |
| Hexose | 51.3 |
| Hexose | 51.6 |
| Disaccharide | 51.7 |
| Hexose | 52.3 |
| Palmitic acid | 52.5 |
| Hexose | 52.7 |
| Hexose | 53.2 |
| Pentitol | 53.7 |
| Pentitol | 53.9 |
| Disaccharide | 54.7 |
| Myo-inositol | 54.9 |
| Margaric acid | 55.5 |
| Disaccharide | 55.9 |
| Hexose | 56.8 |
| Disaccharide | 57.5 |
| Oleic acid | 57.8 |
| Triose-phosphate | 58.1 |
| Stearic acid | 58.3 |
| Sinapic acid | 58.6 |
| Hexose | 58.8 |
| Disaccharide | 59.8 |
| Disaccharide | 60.5 |
| Disaccharide | 60.6 |
| Disaccharide | 61.6 |
| Hexose | 62.1 |
| Disaccharide | 62.2 |
| Dehydroabietic acid | 62.3 |
| Sedoheptulose-7-phosphate | 62.4 |
| Disaccharide | 62.6 |
| Hexose | 62.7 |
| Disaccharide | 63.1 |
| Disaccharide | 63.3 |
| Inositol monophosphate | 63.7 |
| Disaccharide | 63.9 |
| Disaccharide | 64.1 |
| Aucubin | 64.4 |
| Pentose | 65.2 |
| Disaccharide | 66.5 |
| Disaccharide | 67.0 |
| Hexose | 67.1 |
| Adenosine | 67.2 |
| Disaccharide | 67.7 |
| Disaccharide | 69.2 |
| Disaccharide | 69.4 |
| Disaccharide | 70.7 |
| Disaccharide | 72.4 |
| Disaccharide | 72.5 |
| Disaccharide | 72.8 |
| Disaccharide | 73.0 |
| Disaccharide | 73.2 |
| Disaccharide | 73.9 |
| Disaccharide | 77.5 |

**Supplemental table 2. Targeted metabolomics analyses of pennycress embryos at different developmental stages.**

Data are the average ± SD of 3 independent experiments. Numbers highlighted in bold represent a significant difference (*p value <0.05) for a specific metabolite at 2 given comparative developmental stages. Metabolites colored in red and green correspond to glycolysis and TCA cycle, respectively.

| | Analyte | Average ± SD | | | | | | P-value* | | | | |
|---|---|---|---|---|---|---|---|---|---|---|---|---|
| | | 11 DPA | 13 DPA | 15 DPA | 17 DPA | 19 DPA | 21 DPA | 11-13 DPA | 13-15 DPA | 15-17 DPA | 17-19 DPA | 19-21 DPA |
| Sugars and sugar alcohols (pmol/embryo) | Glucose | 1033.2 ± 54.3 | 8360.8 ± 1006.5 | 10558.4 ± 1011.0 | 10065.5 ± 1211.1 | 16743.4 ± 1866.5 | 33479.1 ± 1031.6 | **6.1E-03** | 5.6E-02 | 6.2E-01 | **9.8E-03** | **7.1E-04** |
| | Fructose | 461.9 ± 32.5 | 235.1 ± 27.3 | 138.3 ± 5.6 | 61.8 ± 1.2 | 136.5 ± 21.3 | 153.1 ± 10.5 | **8.7E-04** | **2.2E-02** | **1.2E-03** | **2.6E-02** | 3.1E-01 |
| | Sucrose | 1161.7 ± 130.0 | 9399.6 ± 396.6 | 19417.0 ± 796.2 | 24584.8 ± 230.7 | 29164.8 ± 1061.4 | 34963.7 ± 112.4 | **2.6E-04** | **3.4E-04** | **2.3E-02** | **8.8E-03** | **1.0E-02** |
| | Pentitols | 0.2 ± 0.0 | 4.6 ± 0.2 | 8.7 ± 0.3 | 13.2 ± 0.5 | 15.6 ± 0.2 | 14.7 ± 0.4 | **7.4E-04** | **3.5E-05** | **8.0E-04** | **6.4E-03** | **2.7E-02** |
| | Sorbitol | 127.1 ± 28.1 | 210.4 ± 61.2 | 269.4 ± 26.5 | 368.1 ± 26.2 | 657.0 ± 139.9 | 949.1 ± 281.5 | 1.7E-01 | 1.8E-01 | **1.0E-02** | 6.5E-02 | 3.6E-01 |
| | Erythritol/threitol | 1.6 ± 0.3 | 6.3 ± 0.5 | 12.7 ± 1.0 | 23.7 ± 1.0 | 25.3 ± 1.0 | 27.1 ± 0.4 | **3.0E-04** | **2.4E-03** | **1.7E-04** | 1.3E-01 | 7.4E-02 |
| | Inositol | 237.7 ± 6.1 | 1089.3 ± 67.1 | 1517.2 ± 21.4 | 1354.7 ± 29.3 | 1408.2 ± 99.2 | 844.8 ± 155.5 | **1.6E-03** | **4.6E-03** | **2.1E-03** | 4.5E-01 | **4.5E-03** |
| Amino Acids (pmol/embryo) | Alanine | 1138.8 ± 59.2 | 8266.9 ± 270.2 | 10699.6 ± 692.1 | 12397.1 ± 316.9 | 8669.5 ± 705.0 | 7254.5 ± 537.3 | **2.8E-04** | **1.6E-02** | **3.5E-02** | **4.8E-03** | 5.5E-02 |
| | Arginine | 159.8 ± 16.7 | 1772.8 ± 32.3 | 2609.9 ± 340.2 | 2542.7 ± 202.2 | 1511.6 ± 159.5 | 847.8 ± 127.8 | **4.8E-06** | **5.0E-02** | 7.9E-01 | **2.8E-03** | **5.6E-03** |
| | Asparagine | 707.4 ± 137.1 | 4562.4 ± 292.0 | 2303.6 ± 168.5 | 2312.7 ± 109.1 | 2383.0 ± 170.1 | 2481.7 ± 84.6 | **3.5E-04** | **1.0E-03** | 9.4E-01 | 5.8E-01 | 4.4E-01 |
| | Aspartate | 253.4 ± 24.4 | 1645.0 ± 64.1 | 2751.9 ± 240.7 | 2124.4 ± 93.0 | 2345.1 ± 20.0 | 3543.3 ± 244.4 | **1.6E-04** | **1.1E-02** | **3.3E-02** | **4.9E-02** | **1.3E-02** |
| | Citrulline | 21.2 ± 2.0 | 199.5 ± 6.4 | 309.3 ± 4.5 | 238.0 ± 11.6 | 248.2 ± 12.5 | 356.9 ± 18.4 | **1.3E-04** | **3.9E-05** | **3.9E-03** | 3.6E-01 | **1.9E-03** |
| | Cysteine | 3.4 ± 0.2 | 14.9 ± 2.4 | 24.7 ± 0.2 | 34.5 ± 0.8 | 42.7 ± 2.3 | 65.8 ± 2.4 | **1.3E-02** | **1.9E-02** | **1.3E-03** | **1.7E-02** | **2.7E-04** |
| | GABA | 10.7 ± 0.5 | 30.5 ± 0.3 | 35.3 ± 0.5 | 26.2 ± 1.7 | 26.4 ± 0.2 | 22.5 ± 1.2 | **2.4E-06** | **1.9E-04** | **8.5E-03** | 8.4E-01 | **2.9E-02** |
| | Glutamate | 691.5 ± 104.9 | 5329.3 ± 185.6 | 11239.5 ± 227.3 | 11570.4 ± 365.5 | 10973.7 ± 75.8 | 12245.9 ± 287.9 | **2.7E-05** | **5.9E-06** | 2.7E-01 | 1.0E-01 | **1.2E-02** |
| | Glutamine | 856.0 ± 152.2 | 8132.7 ± 257.3 | 7885.1 ± 494.5 | 13291.9 ± 2482.7 | 12543.5 ± 658.1 | 17422.7 ± 488.7 | **1.5E-05** | 5.0E-01 | 5.9E-02 | 6.6E-01 | **7.5E-04** |
| | Glycine | 47.6 ± 5.7 | 346.1 ± 16.3 | 494.4 ± 7.9 | 370.2 ± 31.2 | 389.5 ± 7.4 | 512.2 ± 17.4 | **3.1E-04** | **9.4E-04** | **1.6E-02** | 4.0E-01 | **2.4E-03** |
| | Histidine | 335.9 ± 26.8 | 890.0 ± 29.8 | 626.3 ± 33.3 | 628.5 ± 4.7 | 618.1 ± 19.5 | 659.2 ± 44.4 | **2.0E-05** | **5.5E-04** | 9.2E-01 | 4.6E-01 | 2.5E-01 |
| | Isoleucine | 21.2 ± 1.7 | 88.5 ± 2.1 | 135.7 ± 7.6 | 141.1 ± 5.8 | 201.9 ± 17.0 | 183.3 ± 2.6 | **3.0E-06** | **5.4E-03** | 3.8E-01 | **1.7E-02** | 2.0E-01 |
| | Leucine | 33.7 ± 1.0 | 107.2 ± 1.2 | 129.5 ± 0.2 | 103.5 ± 8.3 | 169.0 ± 5.6 | 160.9 ± 7.4 | **2.1E-07** | **6.6E-04** | **3.2E-02** | **7.0E-04** | 2.1E-01 |
| | Lysine | 56.5 ± 4.4 | 302.6 ± 2.0 | 279.2 ± 14.4 | 354.3 ± 7.6 | 312.8 ± 6.9 | 348.9 ± 9.2 | **6.9E-06** | 1.0E-01 | **3.9E-03** | **2.3E-03** | **6.8E-03** |
| | Methionine | 9.6 ± 1.2 | 100.7 ± 9.8 | 149.3 ± 1.8 | 154.6 ± 6.8 | 148.4 ± 1.8 | 169.5 ± 11.4 | **3.4E-03** | **1.1E-02** | 3.1E-01 | 2.5E-01 | 8.1E-02 |
| | Hydroxyproline | 8.8 ± 1.4 | 45.0 ± 19.2 | 43.1 ± 1.1 | 44.6 ± 2.1 | 16.1 ± 0.6 | 26.2 ± 0.8 | 8.2E-02 | 8.8E-01 | 3.7E-01 | **9.1E-04** | **1.1E-04** |
| | Ornithine | 3.4 ± 0.0 | 34.2 ± 3.2 | 50.9 ± 2.3 | 43.3 ± 2.1 | 35.6 ± 2.5 | 63.4 ± 4.8 | **2.7E-03** | **2.7E-03** | **3.2E-02** | **2.8E-02** | **2.8E-03** |
| | Phenylalanine | 16.1 ± 0.8 | 71.4 ± 0.8 | 120.6 ± 0.8 | 129.7 ± 2.7 | 160.5 ± 4.6 | 260.7 ± 7.1 | **1.4E-07** | **1.9E-07** | **2.1E-02** | **1.5E-03** | **1.0E-04** |
| | Proline | 966.7 ± 51.6 | 1307.9 ± 45.8 | 1853.0 ± 114.3 | 4171.7 ± 129.8 | 6144.0 ± 9.1 | 8648.0 ± 182.7 | **1.4E-03** | **7.2E-03** | **2.3E-05** | **1.4E-03** | **1.7E-03** |
| | Serine | 365.7 ± 32.7 | 2619.7 ± 48.4 | 3558.7 ± 166.5 | 3524.4 ± 45.5 | 3106.5 ± 30.9 | 3234.6 ± 166.5 | **1.4E-06** | **6.6E-03** | 7.6E-01 | **4.1E-04** | 3.1E-01 |
| | Threonine | 171.8 ± 24.7 | 718.2 ± 17.0 | 1238.0 ± 12.6 | 1186.5 ± 9.2 | 1032.1 ± 22.6 | 1355.8 ± 43.5 | **1.8E-05** | **4.3E-06** | **6.0E-03** | 6.3E-01 | 1.3E-01 |
| | Tyrosine | 18.3 ± 1.1 | 72.8 ± 4.2 | 103.4 ± 1.3 | 104.0 ± 3.6 | 92.5 ± 0.9 | 110.1 ± 8.9 | **1.1E-03** | **3.4E-03** | 8.2E-01 | **2.5E-02** | 7.4E-02 |
| | Valine | 88.0 ± 9.1 | 569.5 ± 14.1 | 591.5 ± 25.1 | 644.8 ± 7.1 | 636.6 ± 25.9 | 873.4 ± 25.7 | **5.0E-06** | **2.7E-01** | **5.7E-02** | **6.5E-01** | **3.6E-04** |
| [illegible]pounds (pmol/embryo) | PGA | 11.1 ± 0.9 | 46.4 ± 1.1 | 56.8 ± 1.6 | 37.7 ± 2.0 | 20.9 ± 2.3 | 20.6 ± 0.5 | **2.2E-06** | **1.5E-03** | **2.8E-04** | **7.9E-04** | 8.6E-01 |
| | 6PG | 31.4 ± 6.1 | 54.3 ± 5.5 | 18.3 ± 0.8 | 26.9 ± 1.0 | 10.5 ± 1.0 | 11.4 ± 1.2 | **8.7E-03** | **6.8E-03** | **4.0E-04** | **3.5E-05** | 3.8E-01 |
| | ADP | 84.4 ± 9.6 | 545.5 ± 10.4 | 639.2 ± 12.9 | 752.2 ± 44.1 | 448.9 ± 32.3 | 451.3 ± 24.3 | **6.3E-07** | **7.6E-04** | **3.8E-02** | **9.9E-04** | 9.2E-01 |
| | AMP | 95.9 ± 4.2 | 255.2 ± 35.2 | 387.0 ± 15.9 | 461.2 ± 18.0 | 540.8 ± 10.2 | 467.2 ± 13.7 | **1.5E-02** | **1.2E-02** | **6.2E-03** | **5.8E-03** | **2.3E-03** |
| | ATP | 17.1 ± 1.0 | 160.2 ± 17.1 | 266.3 ± 26.6 | 189.4 ± 2.1 | 100.2 ± 9.3 | 459.5 ± 22.7 | **4.6E-03** | **7.0E-03** | **3.7E-02** | **2.5E-03** | **3.0E-04** |
| | CDP | 12.7 ± 2.4 | 111.9 ± 1.3 | 128.7 ± 7.3 | 98.2 ± 6.0 | 68.5 ± 2.1 | 75.1 ± 3.1 | **8.1E-06** | 5.4E-02 | **5.7E-03** | **7.7E-03** | **4.7E-02** |
| | CMP | 36.0 ± 0.9 | 58.1 ± 3.3 | 99.9 ± 9.8 | 111.8 ± 4.4 | 134.4 ± 6.5 | 135.9 ± 2.8 | **4.7E-03** | **1.1E-02** | 1.6E-01 | **1.1E-02** | 7.3E-01 |
| | CTP | 3.1 ± 0.7 | 27.9 ± 2.2 | 33.1 ± 1.8 | 21.1 ± 1.1 | 11.9 ± 0.7 | 42.6 ± 2.0 | **1.1E-03** | **3.4E-02** | **1.4E-03** | **7.5E-04** | **5.6E-04** |
| | Deoxyxylulose5P | 2.4 ± 0.3 | 11.1 ± 0.8 | 5.5 ± 0.5 | 5.5 ± 0.2 | 3.3 ± 0.2 | 2.8 ± 0.1 | **1.0E-03** | **1.1E-03** | 8.6E-01 | **1.4E-04** | **1.8E-02** |
| | Sucrose 6P | 1.1 ± 0.1 | 14.4 ± 0.6 | 27.6 ± 0.4 | 32.0 ± 1.5 | 27.3 ± 0.8 | 30.0 ± 0.8 | **4.4E-04** | **2.1E-05** | **3.1E-02** | **1.7E-02** | **1.5E-02** |
| | Trehalose 6P | 1.6 ± 0.3 | 6.9 ± 0.7 | 23.5 ± 0.4 | 22.3 ± 0.1 | 19.8 ± 0.5 | 13.3 ± 0.6 | **1.4E-03** | **1.8E-05** | **3.0E-02** | **1.0E-02** | **1.6E-04** |
| | F1,6bisP | 20.5 ± 2.4 | 72.8 ± 0.9 | 375.4 ± 20.3 | 60.5 ± 2.2 | 97.0 ± 4.6 | 75.3 ± 3.9 | **1.5E-04** | **1.5E-03** | **1.2E-03** | **1.5E-03** | **3.8E-03** |
| | GDP | 21.7 ± 1.6 | 105.5 ± 1.6 | 146.0 ± 0.3 | 164.5 ± 2.7 | 136.5 ± 10.3 | 180.7 ± 16.8 | **3.7E-07** | **3.2E-04** | **6.5E-03** | **3.5E-02** | **2.5E-02** |
| | GTP | 8.3 ± 0.2 | 63.0 ± 2.4 | 104.3 ± 10.7 | 75.8 ± 1.9 | 46.7 ± 2.7 | 160.7 ± 4.3 | **6.3E-04** | 8.3E-01 | 2.2E-01 | **2.4E-04** | **1.3E-05** |
| | GlycerolP | 74.2 ± 5.3 | 461.0 ± 34.8 | 623.5 ± 20.3 | 663.7 ± 53.2 | 405.7 ± 6.1 | 545.7 ± 9.6 | **2.2E-03** | **4.7E-03** | 3.2E-01 | **1.3E-02** | **1.0E-04** |
| | GMP | 41.6 ± 3.2 | 100.2 ± 8.2 | 164.2 ± 2.2 | 186.9 ± 2.4 | 187.7 ± 5.2 | 155.9 ± 5.7 | **2.6E-03** | **3.4E-03** | **2.8E-04** | 8.2E-01 | **2.1E-03** |

| | | | | | | | | | | | | |
|---|---|---|---|---|---|---|---|---|---|---|---|---|
| Phosphorylated com | Fructose 6P | 231.6 ± 44.7 | 1600.3 ± 84.8 | 1715.8 ± 62.1 | 862.9 ± 30.6 | 593.0 ± 18.9 | 566.7 ± 23.0 | **1.3E-04** | 1.4E-01 | **2.7E-04** | **5.8E-04** | 2.0E-01 |
| | Galactose 1P | 47.9 ± 5.2 | 388.5 ± 6.9 | 460.3 ± 3.1 | 463.3 ± 12.3 | 336.4 ± 55.1 | 339.6 ± 23.3 | **6.5E-07** | **7.3E-04** | 7.2E-01 | **6.6E-03** | 8.8E-01 |
| | Glc1P/Man1P | 66.8 ± 7.2 | 457.1 ± 46.4 | 714.6 ± 7.7 | 832.4 ± 35.9 | 669.3 ± 11.9 | 569.3 ± 30.1 | **3.9E-03** | **9.2E-03** | **2.5E-02** | **9.8E-03** | **1.8E-02** |
| | Glucose 6P | 536.4 ± 30.5 | 2304.4 ± 49.5 | 2133.4 ± 70.2 | 1652.4 ± 36.0 | 677.9 ± 58.1 | 885.0 ± 19.6 | **5.6E-06** | **3.1E-02** | **1.9E-03** | **6.8E-05** | **1.7E-02** |
| | Mannose 6P | 33.5 ± 5.0 | 241.6 ± 4.4 | 274.0 ± 2.9 | 271.3 ± 6.0 | 311.7 ± 18.1 | 230.2 ± 6.1 | **8.6E-07** | **9.0E-04** | 5.4E-01 | **4.9E-02** | **9.8E-03** |
| | IMP | 2.6 ± 0.1 | 3.5 ± 0.7 | 5.8 ± 0.3 | 5.7 ± 0.2 | 1.8 ± 0.2 | 1.5 ± 0.0 | 1.9E-01 | **2.1E-02** | 5.4E-01 | **1.1E-05** | 9.8E-02 |
| | PEP | 14.7 ± 2.7 | 64.4 ± 5.0 | 37.5 ± 6.2 | 34.6 ± 1.7 | 22.9 ± 2.0 | 21.4 ± 1.7 | **5.8E-04** | **5.0E-03** | 5.2E-01 | **1.6E-03** | 3.8E-01 |
| | Ribose1P | 2.5 ± 0.4 | 14.7 ± 0.4 | 25.0 ± 0.8 | 25.7 ± 0.5 | 14.6 ± 0.2 | 16.9 ± 1.2 | **3.0E-06** | **6.6E-04** | 3.5E-01 | **1.4E-04** | 7.4E-02 |
| | Ribulose1,5-bisP | 56.4 ± 9.5 | 136.3 ± 10.4 | 321.8 ± 2.2 | 113.9 ± 2.9 | 86.7 ± 2.1 | 94.5 ± 10.4 | **6.3E-04** | **6.7E-04** | **1.4E-07** | **3.0E-04** | 3.0E-01 |
| | Pentose-5Ps | 206.1 ± 22.6 | 544.2 ± 25.6 | 635.6 ± 22.1 | 548.6 ± 32.8 | 441.0 ± 46.5 | 414.8 ± 4.2 | **7.6E-05** | **1.0E-02** | **2.4E-02** | **3.6E-02** | 4.3E-01 |
| | S7P | 81.9 ± 9.3 | 237.2 ± 27.0 | 139.2 ± 4.4 | 121.0 ± 8.2 | 57.3 ± 2.4 | 108.4 ± 3.2 | **6.4E-03** | **2.5E-02** | **4.2E-02** | **3.2E-03** | **4.5E-05** |
| | UDP | 22.7 ± 3.0 | 161.5 ± 9.8 | 320.2 ± 14.3 | 265.1 ± 13.7 | 190.7 ± 4.1 | 219.6 ± 9.3 | **7.4E-04** | **2.0E-04** | **8.6E-03** | **7.2E-03** | **2.0E-02** |
| | UDPGlc | 32.0 ± 1.5 | 469.0 ± 47.1 | 520.9 ± 12.9 | 871.1 ± 55.5 | 1108.4 ± 52.1 | 1112.5 ± 66.5 | **3.8E-03** | 1.9E-01 | **6.1E-03** | **4.0E-02** | **2.0E-03** |
| | UMP | 68.0 ± 3.8 | 441.2 ± 19.3 | 618.2 ± 9.4 | 764.9 ± 15.3 | 707.7 ± 17.8 | 681.2 ± 38.9 | **6.0E-04** | **9.0E-04** | **4.5E-04** | **1.4E-02** | 3.7E-01 |
| | UTP | 4.7 ± 0.8 | 57.7 ± 8.2 | 102.3 ± 1.5 | 104.3 ± 2.4 | 71.4 ± 1.9 | 191.0 ± 7.9 | **7.4E-03** | **9.3E-03** | 3.2E-01 | **7.8E-05** | **8.6E-04** |
| Organic Acids (pmol/embryo) | AKG | 58.9 ± 6.0 | 89.0 ± 26.7 | 97.4 ± 6.7 | 246.9 ± 27.5 | 243.6 ± 7.3 | 119.1 ± 8.6 | 1.8E-01 | 6.5E-01 | **4.6E-02** | 3.4E-01 | **5.3E-05** |
| | cis-aconitate | 8.3 ± 1.3 | 54.1 ± 4.2 | 88.7 ± 0.8 | 99.0 ± 7.8 | 113.0 ± 4.7 | 85.8 ± 4.0 | **1.4E-03** | **3.8E-03** | 1.5E-01 | 6.9E-02 | **1.7E-03** |
| | trans-aconitate | 2.7 ± 0.2 | 6.2 ± 0.6 | 9.1 ± 0.6 | 11.4 ± 0.7 | 12.2 ± 0.9 | 19.4 ± 0.2 | **6.7E-03** | **4.7E-03** | **1.3E-02** | 2.9E-01 | **4.3E-03** |
| | Citrate | 2177.9 ± 87.7 | 11358.3 ± 419.3 | 19227.8 ± 180.7 | 21621.9 ± 1641.5 | 18373.9 ± 295.7 | 11849.6 ± 517.4 | **4.3E-04** | **1.7E-04** | 1.3E-01 | 7.1E-02 | **2.2E-04** |
| | Fumarate | 64.0 ± 3.8 | 127.6 ± 3.7 | 287.7 ± 11.7 | 277.0 ± 15.2 | 306.9 ± 4.4 | 430.0 ± 7.7 | **3.2E-05** | **7.7E-04** | 3.9E-01 | 6.7E-02 | **1.0E-04** |
| | Isocitrate | 23.1 ± 1.7 | 107.2 ± 5.4 | 177.1 ± 10.1 | 146.4 ± 11.3 | 87.5 ± 3.1 | 133.7 ± 10.3 | **5.9E-04** | **1.6E-03** | **2.5E-02** | **8.4E-03** | **1.1E-02** |
| | Malate | 3701.9 ± 114.5 | 8616.7 ± 462.3 | 13253.2 ± 126.4 | 11444.1 ± 127.5 | 11140.9 ± 154.0 | 12965.7 ± 412.7 | **1.8E-03** | **1.9E-03** | **6.3E-05** | 6.1E-02 | **9.4E-03** |
| | Succinate | 574.8 ± 5.0 | 812.5 ± 41.5 | 1548.6 ± 23.0 | 2159.4 ± 175.2 | 1525.5 ± 244.0 | 1713.5 ± 23.3 | **9.2E-03** | **8.5E-05** | **2.5E-02** | **2.6E-02** | 3.1E-01 |
| | Shikimate | 11.7 ± 0.9 | 64.5 ± 7.1 | 109.5 ± 5.1 | 103.6 ± 4.5 | 139.9 ± 1.2 | 129.9 ± 0.9 | **5.5E-03** | **1.4E-03** | 2.1E-01 | **3.3E-03** | **4.2E-04** |

**Supplemental table 3. Targeted metabolomics analyses of pennycress embryos at different developmental stages.**

Data are the average ± SD of 3 independent experiments. Numbers highlighted in bold represent a significant difference (*p value <0.05) for a specific metabolite at 2 given comparative developmental stages.

| | Analyte | Average ± SD | | | | | | P-value* | | | | |
|---|---|---|---|---|---|---|---|---|---|---|---|---|
| | | 11 DPA | 13 DPA | 15 DPA | 17 DPA | 19 DPA | 21 DPA | 11-13 DPA | 13-15 DPA | 15-17 DPA | 17-19 DPA | 19-21 DPA |
| Sugars and sugar alcohols (pmol/mg DW) | Glucose | 34934.4 ± 424.1 | 66935.4 ± 5990.0 | 43410.67± 556.7 | 25120.3 ± 2395.2 | 36838.2±5433.0 | 55229.9± 3351.7 | **2.5E-03** | **2.0E-02** | **4.0E-03** | **2.0E-02** | **7.2E-03** |
| | Fructose | 13756.7 ± 117.3 | 1877.9 ± 51.8 | 529.3 ± 30.8 | 243.7 ± 43.2 | 250.3 ± 9.3 | 279.1 ± 9.8 | **1.5E-06** | **1.9E-05** | **1.2E-03** | 8.2E-01 | **2.2E-02** |
| | Sucrose | 45751.3 ± 13603.7 | 74605.2 ± 2387.2 | 82113.7 ± 1902.2 | 76186.7 ± 3655.9 | 70795.4 ± 2106.3 | 64154.1 ± 3724.4 | 6.3E-02 | **1.4E-02** | 8.8E-02 | 1.1E-01 | 7.0E-02 |
| | Pentitols | 4.0 ± 0.6 | 21.7± 0.5 | 87.5 ± 2.1 | 18.6 ± 0.7 | 18.0± 0.8 | 14.3 ± 1.7 | **4.5E-06** | **3.3E-04** | **1.9E-04** | 4.5E-01 | **4.3E-02** |
| | Sorbitol | 3425.4 ± 681.0 | 1581.9 ± 499.6 | 1313.8 ± 190.6 | 1135.3 ± 113.2 | 1510.5 ± 380.1 | 1711.7 ± 591.8 | **2.3E-02** | 4.6E-01 | 2.5E-01 | 2.2E-01 | 7.2E-01 |
| | Erythritol/threitol | 46.0 ± 7.7 | 64.2± 11.2 | 58.9± 1.5 | 65.7 ± 4.3 | 58.5 ± 3.3 | 54.4 ± 0.8 | **4.8E-02** | 4.2E-01 | 9.7E-02 | 8.6E-02 | 1.6E-01 |
| | Inositol | 6389.7 ± 1154.2 | 7613.4 ± 425.9 | 6770.8 ± 550.0 | 3762.4 ± 233.2 | 3141.0 ±120.9 | 1525.9 ± 186.1 | 2.0E-01 | 1.1E-01 | **1.8E-02** | 1.4E-01 | **5.4E-04** |
| Amino Acids (pmol/mg DW) | Alanine | 42632.7 ± 2656.8 | 51619.4 ± 4039.5 | 52704.3 ± 3081.9 | 36385.9 ± 573.8 | 24987.5 ± 3384.7 | 10932.6 ± 514.1 | **4.0E-02** | 7.3E-01 | **9.8E-03** | **2.5E-02** | **1.7E-02** |
| | Arginine | 4464.3 ± 374.8 | 14408.1 ± 696.2 | 12497.4 ± 207.0 | 7830.2 ± 612.8 | 6837.0 ± 675.2 | 1638.4 ± 1638.4 | **1.8E-04** | **3.3E-02** | **2.8E-03** | 1.3E-01 | **2.2E-03** |
| | Asparagine | 48809.2 ± 13896.9 | 44933.0 ± 2359.8 | 8368.3 ± 51.5 | 5295.0 ± 295.6 | 5683.5 ± 346.8 | 5210.2 ± 215.2 | 6.8E-01 | **1.4E-03** | **2.4E-03** | 2.2E-01 | 1.3E-01 |
| | Aspartate | 9181.3± 361.9 | 12951.5± 992.8 | 13682.6± 527.6 | 7431.0 ± 450.8 | 6980.5 ± 550.9 | 7531.7 ± 160.8 | **1.4E-02** | 3.4E-01 | **1.2E-04** | 3.4E-01 | 2.2E-01 |
| | Citrulline | 713.7 ± 74.2 | 1324.8 ± 272.1 | 1422.3 ± 131.7 | 1199.6 ± 180.2 | 569.1 ± 11.8 | 643.7 ± 35.3 | 5.2E-02 | 6.2E-01 | 1.7E-01 | **2.6E-02** | 5.5E-02 |
| | Cysteine | 120.5 ± 6.3 | 138.7± 14.7 | 113.0 ± 4.4 | 106.0 ± 7.4 | 108.5± 2.6 | 106.5± 4.4 | 1.5E-01 | 8.4E-02 | 2.5E-01 | 6.4E-01 | 5.4E-01 |
| | GABA | 456.6 ± 62.4 | 300.3 ± 31.2 | 175.6± 2.8 | 78.3 ± 2.3 | 68.9 ± 5.8 | 57.0 ± 2.4 | **3.1E-02** | **2.0E-02** | **2.0E-06** | 9.1E-02 | 9.1E-02 |
| | Glutamate | 20692.7 ± 4902.4 | 47608.3 ± 2100.4 | 45539.3 ± 1533.4 | 31886.4 ± 754.2 | 27842.3 ± 2532.9 | 23332.5 ± 1204.2 | **2.5E-02** | 2.5E-01 | **9.5E-04** | 9.9E-02 | 7.3E-02 |
| | Glutamine | 21516.3 ± 6285.0 | 84479.4 ± 7938.2 | 41418.2 ± 3279.8 | 53858.5 ± 2031.1 | 40010.0 ± 4192.4 | 43076.2 ± 4654.9 | **5.5E-04** | **5.0E-03** | **8.5E-03** | **1.6E-02** | 4.4E-01 |
| | Glycine | 2421.3 ± 176.5 | 3242.9 ± 149.2 | 2549.2 ± 61.3 | 1080.7 ± 50.6 | 879.6 ± 22.3 | 781.8 ± 121.7 | **3.9E-03** | **7.5E-03** | **7.9E-06** | **1.0E-02** | 3.0E-01 |
| | Histidine | 10905.7 ± 1002.1 | 8353.2 ± 225.7 | 2870.2 ± 73.3 | 2212.6 ± 153.8 | 1411.2 ± 87.5 | 1075.8 ± 85.6 | **4.2E-02** | **1.8E-04** | **7.9E-03** | **3.5E-03** | **9.0E-03** |
| | Isoleucine | 631.1 ± 106.4 | 849.8 ± 10.2 | 589.0± 3.7 | 391.2 ± 0.6 | 447.2 ± 41.4 | 443.6 ± 56.0 | 6.9E-02 | **1.2E-04** | **8.2E-05** | 1.4E-01 | 9.3E-01 |
| | Leucine | 1183.0± 149.1 | 1037.8 ± 32.1 | 579.1 ± 3.3 | 362.5 ± 21.6 | 359.2 ± 18.9 | 262.3 ± 10.7 | 2.3E-01 | **1.5E-03** | **2.7E-03** | 8.5E-01 | **3.7E-03** |
| | Lysine | 2004.0 ± 61.1 | 2685.5 ± 81.8 | 1620.5 ± 59.8 | 1206.4 ± 146.3 | 774.0 ± 44.9 | 676.0 ± 11.3 | **4.9E-04** | **1.0E-04** | **2.6E-02** | **2.8E-02** | 5.6E-02 |
| | Methionine | 321.1 ± 88.2 | 658.6 ± 73.9 | 729.7 ± 5.7 | 448.5 ± 14.7 | 339.4 ± 12.6 | 315.5 ± 34.4 | **7.7E-03** | 2.4E-01 | **2.2E-04** | **4.9E-04** | 5.9E-01 |
| | Hydroxyproline | 327.1 ± 50.0 | 361.9 ± 76.9 | 177.9 ± 3.1 | 78.4 ± 6.6 | 47.9 ± 8.4 | 45.3 ± 2.0 | 5.5E-01 | 5.3E-02 | **2.3E-04** | **8.8E-03** | 6.5E-01 |
| | Ornithine | 99.5± 20.5 | 287.6 ± 14.7 | 235.6 ± 17.3 | 160.2 ± 32.8 | 144.9 ± 39.2 | 130.9 ± 24.6 | **3.6E-04** | **1.7E-02** | **3.8E-02** | **3.8E-02** | 6.3E-01 |
| | Phenylalanine | 596.2 ± 94.8 | 713.5 ± 3.7 | 556.4 ± 14.9 | 373.9 ± 18.3 | 376.0 ± 16.6 | 448.3 ± 47.1 | 1.7E-01 | **1.9E-03** | **2.3E-04** | 8.9E-01 | 1.0E-01 |
| | Proline | 35481.5 ± 3768.7 | 8842.0 ± 1525.2 | 9563.3 ± 191.9 | 12491.8 ± 307.0 | 13198.3 ± 32.6 | 14811.2 ± 455.0 | **2.6E-03** | 5.0E-01 | **4.3E-04** | 5.6E-02 | **2.5E-02** |
| | Serine | 13715.8 ± 1658.4 | 22978.7± 3069.6 | 19772.5 ± 94.1 | 10645.3 ± 480.8 | 8395.1 ± 297.3 | 7749.7 ± 223.6 | **1.8E-02** | 2.1E-01 | **6.3E-04** | **4.4E-03** | **4.4E-02** |
| | Threonine | 4619.2 ± 463.2 | 5558.7 ± 451.6 | 4615.9 ± 101.1 | 3612.1 ± 54.3 | 2905.5 ± 189.5 | 2744.9 ± 16.5 | 6.6E-02 | 6.2E-02 | **5.5E-04** | **1.7E-02** | 2.8E-01 |
| | Tyrosine | 644.4 ± 89.1 | 689.2 ± 6.5 | 437.7 ± 21.7 | 280.1 ± 8.2 | 285.0 ± 21.7 | 284.0 ± 31.9 | 4.8E-01 | **1.2E-03** | **2.7E-03** | 7.4E-01 | 9.7E-01 |
| | Valine | 3106.5 ± 564.0 | 4842.3 ± 342.5 | 3311.9 ± 101.1 | 1769.6 ± 12.5 | 1705.5 ± 135.5 | 1561.3 ± 103.5 | **1.6E-02** | **1.1E-02** | **1.2E-03** | 5.0E-01 | 2.2E-01 |
| ompounds (pmol/mg DW) | 2-3PGA | 282.4 ± 96.1 | 460.5 ± 6.4 | 238.8 ± 21.0 | 138.3 ± 5.6 | 61.6 ± 8.0 | 43.1 ± 1.1 | 8.4E-02 | **1.5E-03** | **1.0E-02** | **3.3E-04** | 5.4E-02 |
| | 6PG | 306.9 ± 136.1 | 395.8 ± 92.2 | 99.6 ± 3.6 | 80.2 ± 3.8 | 34.8 ± 4.0 | 15.6 ± 0.3 | 4.1E-01 | **3.1E-02** | **3.1E-03** | **1.4E-04** | **1.4E-02** |
| | ADP | 2664.6 ± 240.7 | 3823.4 ± 270.5 | 3124.4 ± 33.5 | 2082.9 ± 196.3 | 1107.6 ± 71.8 | 1016.5± 16.3 | **5.4E-03** | **4.5E-02** | **1.0E-02** | **7.2E-03** | 1.5E-01 |
| | AMP | 1894.0 ± 63.2 | 1903.1 ± 47.4 | 2385.0 ± 258.0 | 1658.3 ± 187.1 | 1265.0 ± 108.1 | 945.1 ± 54.6 | 8.5E-01 | 7.9E-02 | **2.0E-02** | **4.7E-02** | **2.0E-02** |
| | ATP | 638.8 ± 42.6 | 1339.0 ± 171.6 | 1217.2 ± 126.2 | 525.6 ± 18.9 | 253.4 ± 39.0 | 364.6 ± 72.8 | **1.5E-02** | 3.8E-01 | **9.7E-03** | **1.9E-03** | 1.0E-01 |
| | CDP | 386.3 ± 110.3 | 870.1 ± 59.6 | 635.8 ± 13.0 | 317.0 ± 16.9 | 172.9 ± 13.2 | 120.7 ± 12.3 | **6.3E-03** | **1.7E-02** | **2.3E-05** | **4.3E-04** | **7.5E-03** |
| | CMP | 486.5 ± 88.1 | 698.4 ± 23.7 | 780.6 ± 94.9 | 480.0 ± 31.3 | 317.5 ± 13.3 | 246.0 ± 8.8 | **4.5E-02** | 2.7E-01 | **2.3E-02** | **5.4E-03** | **2.6E-03** |
| | CTP | 282.4 ± 14.1 | 254.5 ± 26.3 | 151.0 ± 6.1 | 71.8 ± 6.2 | 27.7 ± 1.1 | 33.3 ± 8.6 | 2.0E-01 | **1.7E-02** | **9.5E-05** | **5.4E-03** | 3.8E-01 |
| | Deoxyxylulose5P | 126.4 ± 1.2 | 108.1 ± 6.1 | 28.3 ± 0.4 | 24.4 ± 5.3 | 8.4 ± 0.6 | 5.9 ± 0.1 | **3.1E-02** | **1.9E-03** | 3.3E-01 | **3.3E-02** | **1.5E-02** |
| | Sucrose 6P | 63.1 ± 2.7 | 112.7 ± 11.2 | 117.6 ± 8.3 | 85.5 ± 4.3 | 68.6 ± 4.0 | 47.2 ± 4.5 | **1.3E-02** | 5.7E-01 | **9.4E-03** | **7.5E-03** | **3.7E-03** |
| | Trehalose 6P | 37.4 ± 9.0 | 76.3 ± 0.4 | 85.7 ± 2.3 | 64.0 ± 1.7 | 45.4 ± 1.2 | 30.6 ± 3.6 | **1.7E-02** | **1.7E-02** | **3.3E-04** | **2.3E-04** | **1.3E-02** |
| | F1,6bisP | 753.8 ± 16.7 | 653.8 ± 15.3 | 1473.5 ± 119.9 | 173.3 ± 6.1 | 116.6 ± 17.6 | 118.6 ± 5.4 | **1.6E-03** | **6.4E-03** | **2.8E-03** | **2.1E-02** | 8.7E-01 |
| | GDP | 754.8 ± 43.3 | 809.6 ± 4.7 | 614.4 ± 5.5 | 466.7 ± 14.6 | 224.3 ± 1.9 | 202.3 ± 19.1 | 1.6E-01 | **1.7E-06** | **1.7E-06** | **1.0E-03** | 1.8E-01 |
| | GTP | 325.0 ± 6.1 | 447.0 ± 34.4 | 469.3 ± 51.7 | 235.6 ± 27.7 | 130.7 ± 6.3 | 145.5 ± 14.3 | **2.3E-02** | 5.7E-01 | **5.8E-03** | **1.8E-02** | 2.1E-01 |
| | GlycerolP | 2544.2 ± 65.7 | 3394.4 ± 291.7 | 3287.0 ± 397.5 | 2172.1 ± 147.8 | 1059.4 ± 24.8 | 981.7 ± 55.2 | **3.2E-02** | 7.3E-01 | **2.8E-02** | **4.8E-03** | 1.2E-01 |
| | GMP | 1266.6 ± 35.4 | 1511.2 ± 81.7 | 932.0 ± 81.3 | 544.8 ± 14.8 | 477.4 ± 43.1 | 324.0 ± 7.9 | **2.2E-02** | **9.6E-04** | **1.2E-02** | 1.0E-01 | **2.2E-02** |
| | Fructose 6P | 15261.9 ± 603.0 | 15242.4 ± 1279.8 | 7338.4 ± 96.4 | 3249.2 ± 140.3 | 1727.7 ± 222.5 | 1231.4 ± 7.6 | 9.8E-01 | **8.3E-03** | **6.8E-06** | **1.3E-03** | 6.1E-02 |

| | | | | | | | | | | | | |
|---|---|---|---|---|---|---|---|---|---|---|---|---|
| Phosphorylated c | Galactose 1P | 2541.9 ± 199.4 | 2903.2 ± 32.3 | 1945.0 ± 9.9 | 1221.8 ± 14.2 | 766.1 ± 36.2 | 527.0 ± 24.7 | 8.5E-02 | **1.3E-04** | **8.8E-07** | **6.2E-04** | **1.3E-03** |
| | Glc1P/Man1P | 3243.4± 643.7 | 3756.1 ± 162.8 | 3149.2 ± 141.3 | 2323.4 ± 112.4 | 1301.1 ± 54.7 | 1174.0 ± 35.4 | 3.0E-01 | **8.6E-03** | **8.9E-03** | **3.6E-04** | **3.5E-02** |
| | Glucose 6P | 25807.7 ± 2146.9 | 18066.7 ± 754.1 | 10411.9 ± 548.0 | 3303.6 ± 425.0 | 3313.7 ± 308.0 | 2198.7 ± 291.1 | **1.6E-02** | **2.5E-04** | **9.0E-05** | 9.8E-01 | **1.0E-02** |
| | Mannose 6P | 1328.6 ± 102.9 | 2128.6 ± 29.4 | 1052.5 ± 21.9 | 973.9 ± 16.7 | 720.1 ± 28.4 | 380.3 ± 13.0 | **3.2E-03** | **2.2E-06** | **9.2E-03** | **6.2E-04** | **4.9E-04** |
| | IMP | 23.8 ± 4.4 | 31.8 ± 5.8 | 30.5 ± 2.7 | 16.9 ± 2.5 | 4.1 ± 0.2 | 3.2 ± 0.6 | 1.4E-01 | 7.5E-01 | **3.0E-03** | **1.2E-02** | 9.3E-02 |
| | PEP | 226.6 ± 76.3 | 469.5 ± 57.5 | 148.5 ± 21.2 | 112.4 ± 9.9 | 55.5 ± 3.7 | 36.6 ± 2.9 | **1.4E-02** | **5.4E-03** | 8.0E-02 | **4.8E-03** | **2.8E-03** |
| | Ribose1P | 124.0 ± 3.2 | 154.4 ± 4.1 | 113.9 ± 3.1 | 75.0 ± 1.2 | 40.7 ± 4.1 | 31.8 ± 3.1 | **7.3E-04** | **2.7E-04** | **6.5E-04** | **2.7E-03** | **4.3E-02** |
| | Ribulose1,5-bisP | 1311.1 ± 273.0 | 1238.9 ± 161.4 | 1328.2 ± 49.0 | 337.7 ± 21.7 | 233.7 ± 28.5 | 154.2 ± 17.8 | 7.2E-01 | 4.4E-01 | **1.3E-04** | **8.8E-03** | **2.1E-02** |
| | Pentose-Ps | 4983.6 ± 119.2 | 4467.4 ± 196.9 | 3420.1 ± 165.1 | 1428.6 ± 16.0 | 864.6 ± 49.2 | 678.8 ± 11.9 | **2.6E-02** | **2.4E-03** | **2.1E-03** | **1.1E-03** | **1.8E-02** |
| | S7P | 2668.6 ± 458.6 | 2480.5 ± 48.4 | 444.1 ± 29.1 | 336.0 ± 35.9 | 277.4 ± 21.4 | 181.3 ± 9.6 | 5.5E-01 | **3.7E-06** | **1.7E-02** | 8.7E-02 | **7.4E-03** |
| | UDP | 1116.6 ± 91.2 | 1565.7 ± 15.6 | 1475.4 ± 87.7 | 818.5 ± 67.5 | 425.6 ± 22.2 | 425.9 ± 22.4 | **1.2E-02** | 2.1E-01 | **6.9E-04** | **5.5E-03** | 9.9E-01 |
| | UDPGlc | 2093.5 ± 306.9 | 4713.0 ± 102.7 | 2484.1 ± 140.3 | 2467.2 ± 55.3 | 1631.0 ± 134.4 | 1098.8 ± 151.3 | **2.1E-03** | **4.8E-05** | 8.6E-01 | **3.5E-03** | **1.1E-02** |
| | UMP | 2139.2 ± 267.5 | 4320.7 ± 186.5 | 2703.2 ± 69.0 | 2262.4 ± 37.3 | 1579.6 ± 103.7 | 1114.4 ± 58.1 | **5.8E-04** | **1.8E-03** | **2.1E-03** | **3.7E-03** | **5.7E-03** |
| | UTP | 430.8 ± 26.3 | 503.8 ± 8.2 | 530.4 ± 58.3 | 322.2 ± 14.4 | 178.1 ± 15.5 | 193.4 ± 15.9 | **3.2E-02** | 5.1E-01 | **2.0E-02** | **3.1E-04** | 3.0E-01 |
| Organic Acids (pmol/mg DW) | AKG | 1841.0 ± 55.1 | 1461.3 ± 358.8 | 468.0 ± 17.5 | 1061.8 ± 26.4 | 540.3 ± 27.5 | 221.0 ± 14.9 | 2.1E-01 | **4.1E-02** | **1.9E-05** | **1.9E-05** | **3.4E-04** |
| | cis-aconitate | 532.1 ± 76.7 | 535.4 ± 12.2 | 353.2 ± 16.4 | 289.9 ± 8.1 | 259.9 ± 2.4 | 156.8 ± 5.7 | 9.5E-01 | **1.7E-04** | **1.0E-02** | **1.7E-02** | **1.9E-04** |
| | trans-aconitate | 49.7 ± 14.3 | 52.3 ± 6.2 | 47.4 ± 5.7 | 26.7 ± 1.4 | 25.7 ± 3.8 | 24.9 ± 2.5 | 7.9E-01 | 3.7E-01 | **2.0E-02** | 6.9E-01 | 7.8E-01 |
| | Citrate | 71619.7 ± 3638.6 | 106054.6 ± 2758.4 | 79166.3 ± 4379.1 | 64838.6 ± 2879.9 | 39604.3 ± 230.9 | 34165.7 ± 2126.6 | **3.0E-04** | **1.8E-03** | **1.3E-02** | **4.1E-03** | **4.6E-02** |
| | Fumarate | 1884.9 ± 239.7 | 1240.1 ± 27.1 | 1451.5 ± 22.7 | 766.6 ± 77.1 | 720.0 ± 7.9 | 680.2 ± 33.1 | **4.2E-02** | **5.8E-04** | **2.3E-03** | 4.0E-01 | 1.7E-01 |
| | Isocitrate | 1186.8 ± 39.1 | 1194.7 ± 70.1 | 810.8 ± 48.6 | 529.3 ± 45.7 | 323.4 ± 68.6 | 218.2 ± 18.0 | 8.8E-01 | **2.3E-03** | **1.9E-03** | **1.7E-02** | 1.1E-01 |
| | Malate | 101861.7 ± 11824.2 | 73661.2 ± 415.8 | 38026.1 ± 3857.9 | 27181.6 ± 427.3 | 24440.6 ± 1497.1 | 21653.8 ± 482.8 | 5.4E-02 | **3.6E-03** | **3.8E-02** | 7.7E-02 | 7.3E-02 |
| | Succinate | 18842.6 ± 291.4 | 7770.6 ± 207.9 | 6637.7 ± 529.7 | 4939.2 ± 672.2 | 3404.2 ± 622.7 | 2711.5 ± 98.8 | **2.2E-06** | 5.1E-02 | **2.9E-02** | **4.4E-02** | 1.9E-01 |
| | Shikimate | 531.1 ± 71.6 | 728.8 ± 44.3 | 554.5 ± 8.6 | 332.6 ± 8.4 | 323.3 ± 5.9 | 257.0 ± 3.1 | **2.2E-02** | **1.8E-02** | **5.7E-06** | 2.0E-01 | **4.2E-04** |
